# Targeted Next‐Generation Sequencing Analysis of 1,000 Individuals with Intellectual Disability

**DOI:** 10.1002/humu.22901

**Published:** 2015-09-30

**Authors:** Detelina Grozeva, Keren Carss, Olivera Spasic‐Boskovic, Maria‐Isabel Tejada, Jozef Gecz, Marie Shaw, Mark Corbett, Eric Haan, Elizabeth Thompson, Kathryn Friend, Zaamin Hussain, Anna Hackett, Michael Field, Alessandra Renieri, Roger Stevenson, Charles Schwartz, James A.B. Floyd, Jamie Bentham, Catherine Cosgrove, Bernard Keavney, Shoumo Bhattacharya, Italian X‐linked Mental Retardation Project, UK10K Consortium, GOLD Consortium, Matthew Hurles, F. Lucy Raymond

**Affiliations:** ^1^ Department of Medical GeneticsCambridge Institute for Medical ResearchUniversity of CambridgeCambridgeCB2 0XYUnited Kingdom; ^2^ The Wellcome Trust Sanger InstituteWellcome Trust Genome CampusHinxtonCambridgeCB10 1SAUnited Kingdom; ^3^ Department of HaematologyUniversity of CambridgeCambridgeCB2 0PTUnited Kingdom; ^4^ East Anglian Medical Genetics ServiceAddenbrooke's HospitalCambridgeCB2 0QQUnited Kingdom; ^5^ Molecular Genetics LaboratoryGenetics ServiceCruces University HospitalBioCruces Health Research InstituteBarakaldo‐Bizkaia48903Spain; ^6^ Centre for Biomedical Research on Rare Diseases (CIBERER)Madrid28029Spain; ^7^ Department of Paediatrics and Robinson Research InstituteThe University of AdelaideAdelaideSouth Australia5006Australia; ^8^ SA PathologyWomen's and Children's HospitalAdelaideSouth Australia5006Australia; ^9^ Genetics of Learning Disability ServiceHunter GeneticsWaratahNew South Wales2298Australia; ^10^ Medical GeneticsUniversity of SienaSiena53100Italy; ^11^ Genetica MedicaAzienda Ospedaliera Universitaria SeneseSiena53100Italy; ^12^ Greenwood Genetic CenterGreenwood, South Carolina29646; ^13^ The Genome CentreJohn Vane Science CentreQueen Mary University of LondonCharterhouse SquareLondonEC1M 6BQUnited Kingdom; ^14^ Department of Cardiovascular MedicineUniversity of OxfordWellcome Trust Centre for Human GeneticsOxfordOX3 7BNUnited Kingdom; ^15^ Cardiovascular Research GroupInstitute of Cardiovascular SciencesUniversity of ManchesterManchesterM13 9NTUnited Kingdom

**Keywords:** intellectual disability, next‐generation sequencing, Mendelian disease, developmental delay

## Abstract

To identify genetic causes of intellectual disability (ID), we screened a cohort of 986 individuals with moderate to severe ID for variants in 565 known or candidate ID‐associated genes using targeted next‐generation sequencing. Likely pathogenic rare variants were found in ∼11% of the cases (113 variants in 107/986 individuals: ∼8% of the individuals had a likely pathogenic loss‐of‐function [LoF] variant, whereas ∼3% had a known pathogenic missense variant). Variants in *SETD5*, *ATRX*, *CUL4B*, *MECP2*, and *ARID1B* were the most common causes of ID. This study assessed the value of sequencing a cohort of probands to provide a molecular diagnosis of ID, without the availability of DNA from both parents for de novo sequence analysis. This modeling is clinically relevant as 28% of all UK families with dependent children are single parent households. In conclusion, to diagnose patients with ID in the absence of parental DNA, we recommend investigation of all LoF variants in known genes that cause ID and assessment of a limited list of proven pathogenic missense variants in these genes. This will provide 11% additional diagnostic yield beyond the 10%–15% yield from array CGH alone.

## Introduction

As the quality of antenatal and postnatal care improves, the genetic contribution to intellectual disability (ID) becomes ever more significant and has emerged as the commonest phenotypic manifestation of a constitutional genomic abnormality [Ropers, 2010]. ID is prevalent, despite severely reducing fecundity, due to a high de novo mutation rate in a wide range of genes leading to rare Mendelian diseases [Vissers et al., 2010]. Strategies to identify novel disease causing genes have successfully used de novo trio analysis to compare the proband's genome against the parental genomes with a diagnostic rate of 15%–40% [de Ligt et al., 2012; Rauch et al., 2012; Gilissen et al., 2014; Hamdan et al., 2014; The Deciphering Developmental Disorders Study, 2014] and a further 10%–20% diagnostic rate using a homozygosity mapping or biallelic variant analysis strategy [Musante and Ropers, 2014].

In single‐parent households, which are 28% of households with dependent children in the UK, as per the 2013 report of Office of National Statistics, the lack of readily available DNA from both parents excludes the use of a proportion of these families in trio analysis. Nevertheless, analysis of singletons allows detection of novel genes by analysis of a large number of cases in a replication cohort. This strategy has been successfully used to identify novel ID‐associated genes where multiple independent loss‐of‐function (LoF) variants cause disease (e.g., *SETD5*; MIM #615743) [Grozeva et al., 2014], although the interpretation of rare missense variants is challenging and uncertain.

We have used a large‐scale cohort of 986 cases with moderate to severe ID to inform a strategy and likely diagnostic yield in non‐nuclear families using our current knowledge of the Mendelian causes of ID. More specifically, we undertook a targeted panel‐based next‐generation sequencing (NGS) study to identify rare coding variants in 565 selected known and candidate ID‐associated genes. The aims of the study were twofold: (i) to estimate the contribution of variants in known ID‐associated genes to disease, and (ii) to identify novel ID‐associated genes.

## Materials and Methods

### UK10K Cohort

The ID cohort consisted of DNA samples from 996 patients with moderate‐to‐severe ID from the UK, Australia, Spain, USA, and Italy with majority of the individuals recruited from the UK (70%). The appropriate research ethical approval was obtained (IRAS 03/0/014), and parents or guardians provided written informed consent. A clinical geneticist had assessed the individuals and the cause of the ID was unknown. The cases had previously been tested negative by routine diagnostic approaches (i.e., CGH microarray analysis at 500 kb resolution, fragile X [MIM #300624], methylation status of Prader Willi [MIM #176270]/Angelman syndrome [MIM #105830]). The gender ratio is 93.8% male and 6.2% female as the sample was originally collected to assess the contribution of variants in X‐linked disease genes to ID. The ID cohort was a subset of a large replication study of seven rare diseases and comprised a total of 2,812 individuals who were investigated within the UK10K Project Rare Disease replication cohort (http://www.uk10k.org/). The studied phenotypes were 996 cases with ID, 905 cases with congenital heart disease (CHD); 911 cases comprising ciliopathy, coloboma, neuromuscular disease, severe insulin resistance, congenital thyroid disease, and 13 internal technical control samples were also included for comparison. The analysis reported here only uses results from the ID and CHD cohorts.

### Panel of ID‐Associated Genes

A list of known and candidate genes previously associated with ID was compiled on the basis of current literature, in‐house data, and sequence homology to genes previously implicated in ID. The full list of the studied 565 genes can be found in Supp. Table S1. In total, the panel consisted of 253 known and 312 candidate ID‐associated genes. To annotate the genes as either known or candidate, we combined information from DDDG2P list, Gilissen et al. (2014) and in‐house manual curation for a small proportion of the genes (DDDG2P list is available at http://decipher.sanger.ac.uk/ddd/ddd_genes). To be considered as a known cause for ID, the gene had to be annotated as known in either the DDDG2P list or the Gilissen et al. (2014) study. The rest of the genes were considered candidate [Gilissen et al., 2014; The Deciphering Developmental Disorders Study, 2014].

### NGS Experiment

Initially, 1,013 DNA samples from probands with ID were submitted for the NGS analysis, from which 17 samples did not meet the DNA quality criteria, and a further 10 did not meet downstream quality control of variant data. The DNA samples were whole‐genome amplified (GenomiPhi V2 DNA Amplification Kit; GE Healthcare, Little Chalfont, Buckinghamshire, United Kingdom). A custom‐based targeted Agilent SureSelect pull‐down array was created (Agilent Technologies, Santa Clara, California) and the Illumina HiSeq 2000 platform was used to sequence the exons of the targeted regions. Full details of the methods have been previously published [Grozeva et al., 2014]. The data are based on the GRCh37/hg19 version of the reference genome. The variants were filtered based on frequency (MAF<1%) in 1000 Genomes, the UK10K twins cohort, the NHLBI Exome Sequencing Project (Exome Variant Server [EVS], http://evs.gs.washington.edu/EVS/), a cohort of 2,172 in‐house whole‐exome controls and the UK10K rare replication cohort itself (including all phenotypes). We analyzed variants with predicted functional consequence on the protein (i.e., nonsynonymous, inframe indels, and stop codon loss) and variants predicted to cause LoF of the protein (i.e., nonsense, frameshift, and essential splice‐site variants). Functional annotations were added with the Ensembl Variant Effect Predictor 2.8 against Ensembl 70 with the annotation and the corresponding protein consequence reflecting the most severe impact among the gene transcripts (Supp. Table S2) [McLaren et al., 2010]. Where an individual had more than one rare coding heterozygous mutation in the same gene, this was flagged as a potential compound heterozygote. Variants were assessed in clinical databases (e.g., the HGMD [Stenson et al., 2014]); evidence was sought for involvement of the gene in ID (i.e., known or candidate ID gene), whether the variants were present in the canonical gene transcript corresponding to the GenBank mRNA NCBI Reference Sequence, consistency with the expected mode of inheritance, and the clinical phenotype of the individual. A proportion of the likely pathogenic variants was either confirmed by Sanger sequence analysis or by whole‐exome sequence analysis using Agilent SureSelect Human All Exon V5 (Agilent Technologies) from an independent sample of nonamplified DNA from the probands.

### Principal Component Analysis and Cohort Allelic Sums Test

Principal component analysis (PCA) was performed using the R package SNPRelate [Zheng et al., 2012]. We first identified a list of appropriate SNPs from the UK10K samples, which were high‐quality, biallelic, polymorphic, common (minor allele frequency ≥ 0.05), and not in high‐linkage disequilibrium with each other. We next performed PCA on the UK10K samples, along with a subset of unrelated HapMap3.3 samples, using the SNPs identified [Altshuler et al., 2010]. To assess whether there was an enrichment of variants in ID‐associated genes in cases with ID as compared with the CHD sample set, we used the cohort allelic sums test (CAST) [Morgenthaler and Thilly, 2007].

## Results

### Overview of ID Cohort

Overall, we observed 9,015 coding nonsynonymous variants in known and candidate genes passing the quality control and frequency filters (MAF<1%) in 996 individuals. Ten individuals had more than 30 variants per person, whereas the mean number of variants per person was nine (Supp. Fig. S1). These individuals were excluded from the subsequent analysis, as they were outliers from the variant distribution and the variants were likely to be due to technical artifacts. Following this, the total observed nonsynonymous coding variants was 8,466 in 986 individuals (Supp. Table S2). Of these, 8,011 were classified as missense, inframe indels, or stop codon losses. The remaining, 455, were classified as LoF (184 were nonsense, 161 were frameshift, 66 were essential splice donor, and 44 were essential splice acceptor variants, respectively). The mean number of LoF variants per person was 0.46, whereas the mean number of missense variants per person was eight (Supp. Figs. S2 and S3). A subset of variants was experimentally validated with an independent platform. Seventy nine out of the 82 (96%) tested variants were independently confirmed.

### LoF Variants in Known ID‐Associated Genes

The primary aim of this study was to estimate the contribution of LoF and missense variants in known ID‐associated genes to disease. Altogether, LoF variants considered likely to cause disease were observed in 44 genes out of the 253 known ID‐associated genes, in the ID cohort. This represented 77 variants from the observed 455 LoF variants, which were assessed as likely to be the cause of disease in 77 individuals (Table 1; Supp. Table S3). Therefore, the overall diagnostic rate considering LoF variants in known genes alone is ∼8% of the whole sample. Approximately 17% (13/77) of the LoF variants in known genes are present in HGMD (Professional version 2014.3) [Stenson et al., 2014], whereas the remainder are novel observations (i.e., variants at these genomic positions have not been observed in public databases or have been published previously), suggesting that the full extent of the variation in the recently identified genes that leads to ID is not fully documented as yet [Tarpey et al., 2009; Tennessen et al., 2012].

**Table 1:** Genes with LoF Variants Classified as Likely Pathogenic, Ranked According to the Number of Observed Variants

Autosomal‐dominant inheritance		X‐linked inheritance		Recessive inheritance	
Gene	*N* cases	Gene	*N* cases	Gene	*N* cases
*SETD5*	7	*ATRX*	6	*HEXA*	1 (comp. het. –LoF and a missense variant)
*ARID1B*	4	*CUL4B*	5	*AGA*	1 (homoz.)
*GRIN2B*	2	*IL1RAPL1*	3	*HGSNAT*	1 (comp. het.‐ LoF [homoz.] and a missense variant)
*SCN2A*	2	*BRWD3*	2	*PAH*	1 (homoz.)
*CHD7*	2	*OPHN1*	2		
*CTNNB1*	2	*PQBP1*	2		
*KAT6B*	2	*SLC9A6*	2		
*TCF4*	2	*UPF3B*	2		
*KANK1*	2	*ZDHHC9*	2		
*ASXL1*	1	*NLGN4X*	1		
*MLL2*	1	*ACSL4*	1		
*SCN8A*	1	*AFF2*	1		
*EHMT1*	1	*GPC3*	1		
*FOXP1*	1	*KDM5C*	1		
*MEF2C*	1	*MAOA*	1		
*NSD1*	1	*OFD1*	1		
*PAX6*	1	*PTCHD1*	1		
*PTEN*	1	*TSPAN7*	1		
*SHANK2*	1	*USP9X*	1		
*SETBP1*	1				
*UBE3A*	1				

N, number; comp. het., compound heterozygous variants; homoz., homozygous variant.

Subsequently, we assessed whether these variants are present in a recently released large dataset of anonymized exome data from a range of clinical phenotypes including neuropsychiatric disorders (Exome Aggregation Consortium (ExAC), Cambridge, MA; URL: http://exac.broadinstitute.org; accessed November 2014, Supp. Table S3). Of the 77 variants considered potentially pathogenic, only five were seen in the ExAC data (with low frequencies ≤0.002%), four of which cause autosomal‐recessive disease ([*PAH*; MIM #612349], [*HGSNAT*; MIM #610453], [*HEXA*; MIM #606869], and (*AGA*; MIM #613228]) and as expected some of these variants were seen in a heterozygous state in the control ExAC population at low frequencies (four alleles in ∼122,000 sequenced alleles) [Tabor et al., 2014]. One variant in *KANK1* (MIM #607704) (considered autosomal‐dominant gene), which was annotated as likely pathogenic, was observed twice in the ExAC data out of the studied ∼63,000 individuals. These variants remain likely to be pathogenic in our cases but as the phenotype of the ExAC cases is not recorded, the significance of the ExAC data is yet unclear.

### Enrichment Analysis Yields Additional Insights into Disease Etiology

In order to estimate the sensitivity with which we have identified causative LoF variants, and to gain additional insights into disease etiology, we assessed the enrichment of LoF variants in sequenced ID‐associated genes in our cohort, compared with a comparison dataset, using the CAST. For comparison, we used the CHD cohort, which was one of the seven rare disease cohorts in the UK10K replication study and was sequenced in the same experimental pipeline. The ID and CHD cohorts are of similar size, have minimal overlap in phenotypic spectra, and have no qualitative difference in population structure (Supp. Fig. S4).

**Figure 1:**
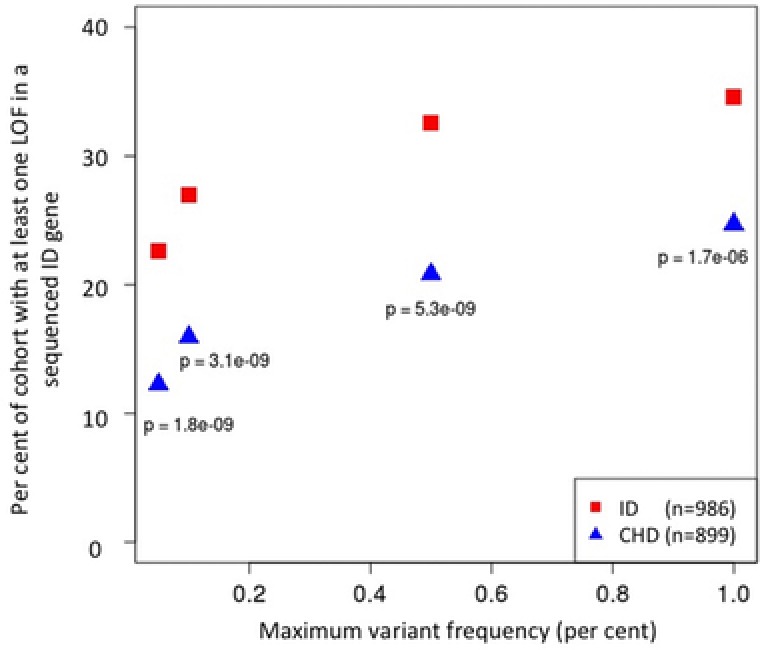
Patients with ID have an enrichment of LoF variants in sequenced ID‐associated genes compared with the CHD cohort. Numbers in key show number of samples. *P* values were calculated by one‐tailed Fisher's exact test.

Of the 986 ID samples, 341 (34.6%) had at least one rare LoF variant in one of the known or candidate 565 sequenced ID‐associated genes, compared with 222/899 (24.7%) in CHD (Fig. 1). This represents a highly significant enrichment (*P* = 1.7×10^−6^). The difference between the cohorts suggests that ∼10% of the ID cases in this cohort are caused by LoF variants in the sequenced genes. This is similar to our actual diagnostic rate of ∼8% for LoF variants, suggesting that we have identified causal LoFs with high sensitivity. We next applied more stringent internal variant frequency filters of 0.5%, 0.1%, and 0.05%, the latter of which leaves unique variants only. Regardless of the filter, the difference between the cohorts is maintained, indicating that the majority of the LoF variants that cause ID in this cohort are unique variants. The large majority of LoF variants in the CHD cohort are unlikely to be pathogenic. For example, in 125 patients, the variant is present in an external control or in another individual within the dataset. In further 84 patients, the variants are either in a candidate gene or in a heterozygous state but in a known gene for ID, implicated in autosomal‐recessive disease.

Next, the LoF variants were categorized according to chromosome, variant type, whether the sequenced ID‐associated gene is known to cause disease or is a candidate gene where the evidence for disease causation is more limited, and whether abnormalities in the gene cause disease according to a biallelic or a monoallelic mode of inheritance. We performed the CAST test to evaluate the degree of enrichment of each of these categories of unique LoF variants in the ID cohort (Table 2).

**Table 2:** Enrichment of Unique LoF Variants in the ID Cohort, Split by Category

Gene category		Variant type	Frequency ID	Frequency CHD	*P* value
Autosome or PAR	Known monoallelic *78*	LoF SNVs	42/986 (4.26%)	6/899 (0.67%)	1.89×10^−7a^
		LoF indels	13/986 (1.32%)	7/899 (0.78%)	0.180
		Synonymous	394/986 (39.96%)	357/899 (39.71%)	0.475
	Known biallelic *86*	LoF SNVs	43/986 (4.36%)	26/899 (2.89%)	0.057
		LoF indels	23/986 (2.33%)	13/899 (1.45%)	0.108
		Synonymous	294/986 (29.82%)	258/899 (28.7%)	0.315
	Candidate *162*	LoF SNVs	52/986 (5.27%)	22/899 (2.45%)	0.001^a^
		LoF indels	26/986 (2.64%)	23/899 (2.56%)	0.516
		Synonymous	450/986 (45.64%)	400/899 (44.49%)	0.325
X chromosome (males only)	Known *89*	LoF SNVs	17/925 (1.84%)	0/466 (0%)	9.25×10^−4a^
		LoF indels	14/925 (1.51%)	0/466 (0%)	0.003^a^
		Synonymous	121/925 (22.92%)	41/466 (8.8%)	0.0108
	Candidate *150*	LoF SNVs	11/925 (1.19%)	2/466 (0.43%)	0.135
		LoF indels	4/925 (0.43%)	1/466 (0.21%)	0.457
		Synonymous	179/925 (19.35%)	92/466 (19.74%)	0.599

The numerator in the “Frequency ID” and “Frequency CHD” columns show the number of samples in each cohort that have one of more unique variant of the category indicated. Synonymous variants are included as controls. Variants of all genotypes are included. The number of genes in each category is given in italics.

PAR, pseudo‐autosomal region; SNV, single‐nucleotide variant; LoF, loss–of‐function; ID, intellectual disability cohort; CHD, congenital heart disease cohort. *P* values calculated using Fisher's exact test.

^a^Statistically significant after Bonferroni correction for multiple testing (*P* value threshold: 0.005).

LoF single‐nucleotide variants (SNVs) in autosomal, known ID‐associated genes with monoallelic mode of inheritance are significantly enriched in the ID cohort (*P* = 1.89×10^−7^). In contrast, we identified no significant enrichment in known ID‐associated genes with biallelic mode of inheritance (*P* = 0.057). This is consistent with studies showing that de novo LoF variants are a particularly frequent cause of ID in Western populations, whereas in populations with high rate of consanguinity, variants in genes implicated in autosomal‐recessive diseases make a significant contribution [Najmabadi et al., 2011; de Ligt et al., 2012; Rauch et al., 2012; Gilissen et al., 2014; Hamdan et al., 2014; Musante and Ropers, 2014].

Interestingly, in contrast to autosomal candidate genes (*P* = 0.001), we identified no significant enrichment on SNVs in X‐linked candidate genes (*P* = 0.135). This suggests that, compared with autosomes, a higher proportion of ID‐associated genes on the X chromosome has already been identified. Importantly, as expected, there is no significant enrichment of synonymous variants in sequenced ID‐associated genes in the ID cohort compared with CHD (Table 2).

### Missense Variants in Known Genes

In total, 8,011 high‐quality rare missense variants (minor allele frequency<1%) were observed in the ID cohort. Assigning pathogenicity to missense variants is inherently more complex than for LoF variants as a lower proportion of missense variants affect protein function. This is compounded when the inheritance of the missense variant is unclear without parental analysis. Xue et al. (2012) showed that potentially every individual carries large numbers of rare missense substitutions in their genome [Xue et al., 2012]. As the studied ID cohort consisted of affected individuals, without parents, we only analyzed the 400 missense variants that had been reported previously to lead to disease, based on the HGMD database (Professional release 2014.3) [Stenson et al., 2003; Stenson et al., 2014]. After manual curation of these 400 missense variants, only 36 (9%) were classified as likely pathogenic in 33 individuals in this study (Table 3; Supp. Table S4).

**Table 3:** Genes with Missense Variants Classified as Likely Pathogenic, Ranked According to the Number of Observed Variants

Autosomal‐dominant inheritance		X‐linked inheritance		Recessive inheritance	
Gene	*N* cases	Gene	*N* cases	Gene	*N* cases
*KRAS*	2	*MECP2*	5	*DHCR7*	1 (comp. het.)
*SLC2A1*	2	*ATRX*	2		
*ARID1B*	1	*MED12*	2		
*CHD7*	1	*DMD*	1		
*GRIN2A*	1	*GRIA3*	1		
*CNTNAP2*	1	*PAK3*	1		
*HRAS*	1	*SMS*	1		
*KCNQ3*	1	*ZDHHC9*	1		
*MAP2K1*	1				
*NSD1*	1				
*PRRT2*	1				
*PTEN*	1				
*SHOC2*	1				
*STXBP1*	1				
*TUBA1A*	1				

*N*, number; comp. het., compound heterozygous variants.

The manual filtering of the 400 variants reported in HGMD included removing the following: (i) variants in a gene that is not reported as causing ID (i.e., candidate genes); (ii) the presence of the variant in public control databases (NHLBI Exome Sequencing Project [EVS, http://evs.gs.washington.edu/EVS/]), or internal control sets, except where the gene causes autosomal‐recessive disease; (iii) variants in genes with autosomal‐recessive inheritance if only one abnormal allele was observed; (iv) NCBI PubMed/literature search excluded the variant as pathogenic; (v) assessment if other rare variants (i.e., LoF variants) in the studied individual were more likely to explain the ID; (vii) pedigree and phenotypic information predicted a specific mode of inheritance.

In total, 26 genes from the 253 known ID‐associated genes (10%) had a likely pathogenic missense variant (Table 1; Table 3; Supp. Table S4). Overall, 33 individuals had a potentially disease‐causing missense variant. The total number of observed likely pathogenic variants was 36, as one individual had compound heterozygous missense variants in *DHCR7* (MIM #602858), another individual had two missense variants that were potentially pathogenic (in *PRRT2* [MIM #614386] and *MECP2* [MIM #300005]), whereas another had two missense variants in *SLC2A1* (MIM #138140).

We observed rare missense variants in genes with recessive inheritance in the ExAC data (*DHCR7* and *HGSNAT*). With respect to *DHCR7*, one of the observed alleles was seen with relatively high frequency in Asian and African controls in the ExAC data (195 alleles/∼110,000 alleles in total). As the studied proband in this cohort was also of South Asian origin, it is possible that the variant is a founder‐specific risk factor for Smith‐Lemli‐Opitz syndrome (MIM #270400) or an incidental nonpathogenic variant [Xue et al., 2012]. In addition, some of the variants observed in *KRAS* (MIM #190070), *NSD1* (MIM #606681), *SLC2A1* (MIM #138140), and *CNTNAP2* (MIM #604569) were each observed once in ∼100,000 sequenced alleles in the ExAC data (Supp. Table S4). The clinical significance of these variants is therefore uncertain, although these variants in the cases were clinically plausible based on information from the phenotype case notes.

We applied the CAST test to unique missense variants in an attempt to estimate the possible contribution of causal missense variants in our cohort. To enrich for damaging missense variants, we applied stringent criteria, including only variants that are predicted to be damaging by each of four scores of predicted damage (PolyPhen2, SIFT, Condel, and CADD). The used cut off scores were the following: PolyPhen2 score >0.9, SIFT <0.06, Condel >0.47, and CADD score (Phred called) >20 [Clifford et al., 2004; Kumar et al., 2009; Adzhubei et al., 2010; Gonzalez‐Perez and Lopez‐Bigas, 2011; Sim et al., 2012; Kircher et al., 2014], and that are absent from reference datasets. We excluded the samples for which a clearly causal LoF variant had been identified. 284/900 (31.6%) of the ID cohort had at least one unique, predicted damaging, missense variant in a sequenced ID‐associated gene, compared with 245/899 (27.3%) in the CHD cohort. In contrast to LoF variants, this represents only a slightly significant enrichment (*P* = 0.025), emphasizing that while missense variants are likely to be the cause of ID in some cases, their interpretation is much more challenging than that of LoF variants, as has previously been shown [Grimm et al., 2015], even following stringent preselection for likely damaging variants.

### LoF Variants in Candidate ID‐Associated Genes

The second aim of this study was to identify novel ID‐associated genes, by assessing LoF variants in the 312 candidate ID‐associated genes, to determine whether there was sufficient evidence to confirm the association of any of these candidates with disease. We previously successfully employed this strategy to identify *SETD5* as an ID‐associated gene [Grozeva et al., 2014]. Here, we sought to determine whether any other candidate ID‐associated genes could similarly be confirmed. We observed LoF variants in eight candidate ID‐associated genes (Table 4; Supp. Table S3).

**Table 4:** Candidate ID‐Associated Genes with LoF Variants and Previous Evidence for Their Involvement in ID

Gene	*N* cases in this study	*N* cases previous NGS studies (total number of studied individuals, disorder)	*N* LoF variants in the genes in EVS (*N* = 6503)/ExAC (*N* = 61486)	Mode of inheritance	Complementary supporting evidence	Residual intolerance score (percentile) [Petrovski et al., 2013]	Article
*ZMYM6*	2	1 LoF (100 trios, ID)	3/23	AD		−0.33 [31]	de Ligt et al. (2012)
*PHF10*	2	1 Lof in an unaffected sibling (343 trios, autism)	2/16	AD	6q27 region, one of four genes in the smallest region of overlap, structural brain abnormalities	−0.43 [25]	Iossifov et al. (2012); Peddibhotla et al. (2014)
*PHIP*	1	1 LoF (100 trios, ID)	2/10	AD		−1.23 [5.5]	de Ligt et al. (2012)
*ASH1L*	1	1 missense (100 trios, ID); 6 missense (765 cases, replication, ID)	1/7	AD		−1.80 [2.2]	de Ligt et al. (2012)
*WAC*	1	1 LoF (100 trios, ID)	0/3	AD	Eight deletions in 29,085 individuals with DD	−0.87 [10]	Coe et al. (2014); de Ligt et al. (2012)
*FAM120C*	1		0/6	X‐linked	Xp11.22 region, one of three genes in the smallest region of overlap, autism, ID	−0.14 [44]	De Wolf et al. (2014); Holden and Raymond (2003); Qiao et al. (2008)
*WNK3*	1		0/1	X‐linked	Xp11.22 region, one of three genes in the smallest region of overlap, autism, ID	−1.33 [5]	Qiao et al. (2008)
*TRMT1*	1 (comp. het.)	1 LoF (136 families, ARID)	6/32	AR		−0.80 [13]	Najmabadi et al. (2011)

ARID, autosomal‐recessive ID; AD, autosomal dominant; AR, autosomal recessive; comp. het., compound heterozygous; N, number; EVS, data from the NHLBI Exome Sequencing Project (Exome Variant Server, http://evs.gs.washington.edu/EVS/); ExAC, data from the Exome Aggregation Consortium ([ExAC], Cambridge, MA; URL: http://exac.broadinstitute.org; accessed November 2014).

We did not observe sufficient number of individuals with LoF variants in any of these candidate genes in our study alone to justify reassigning the status of the gene from a candidate to a known gene status. However, the reporting of novel variants in these genes provides additional evidence that these are likely to be ID‐associated genes. We collated the current evidence for pathogenicity for these genes and with the addition of data from this study, it is increasingly plausible that LoF variants in *ASH1L* (MIM #607999), *ZMYM6* (MIM #613567), *PHF10* (MIM #613069), *PHIP* (MIM #612870), and *WAC* (MIM #615049) are sufficient to cause disease in the affected individuals; *FAM120C* (MIM #300741) and *WNK3* (MIM #300358) are increasingly likely X‐linked ID genes, and further rare variants in *TRMT1* (MIM #611669) increase the evidence that it is an autosomal‐recessive ID gene (Table 4). Additional evidence, albeit indirect, is that no other potentially pathogenic variants were observed in any of these individuals in the remaining known ID‐associated genes within the study, although a comprehensive exome analysis was not performed. Moreover, all of the candidate genes had negative intolerance scores that suggest intolerance to functional variation and indicate purifying selection [Petrovski et al., 2013].

Our observation of further rare variants in these candidate genes provides additional support for the role of these genes in ID. Furthermore, all of these variants were unique to the cohort and were absent from the control populations (including ∼122,000 alleles from the ExAC data). Nonetheless, the identification of additional cases with rare likely pathogenic variants is required in order to confirm that these genes are disease causing.

## Discussion

We performed targeted NGS analysis of 986 cases with ID through a panel of 565 genes, which were either known to cause disease or were candidate ID‐associated genes. We observed 8,466 rare high‐quality variants in 986 individuals with moderate‐to‐severe ID. On average, we observed 0.5 LoF rare variants and eight missense rare variants per person. Rare variants were found in 233 genes out of the 253 known ID‐associated genes, illustrating that rarity of a variant in such a genetically heterogeneous disorder is insufficient to invoke causality.

The first aim of this study was to estimate the contribution of variants in known ID‐associated genes to disease. After manual curation of LoF and missense variants in known genes, we were able to provide a diagnosis for 107/986, ∼11% of cases (77 individuals [∼8%] had a LoF variant and 30 [∼3%] cases had a causal missense variant, whereas three individuals had both a LoF and a missense variant and hence were counted once). For LoF variants, the predicted and identified number of causative variants between the enrichment analysis and the manual curation is consistent, but for missense variants the analysis was significantly limited by the lack of parental DNA samples for de novo trio analysis to distinguish rare familial from de novo variants. It is therefore likely that there are additional causal missense variants in this cohort that we were unable to confidently identify as such.

5% of the studied cohort had a disease‐causing variant in a known X‐linked ID gene. This rate is similar to the rate observed in other recent studies in ID, with the contribution of disease‐causing variants on the X chromosome ranging from 2.5% to 17% [Vissers et al., 2010; de Ligt et al., 2012; Rauch et al., 2012; Gilissen et al., 2014; Hamdan et al., 2014; Redin et al., 2014; The Deciphering Developmental Disorders Study, 2014; Yang et al., 2014].

The most commonly observed genes with LoF variants were *SETD5*, *ATRX* (MIM #300032), *CUL4B* (MIM #300304), and *ARID1B* (MIM #614556). The cohort was ascertained due to the presence of ID and is relatively biased against recruitment of cases with a clearly syndromic form of disease. Despite this, the yield of rare variants in genes normally associated with a distinct syndromic phenotype was surprising (e.g., in *TCF4* [MIM #602272] *CHD7* [MIM #608892], *ARID1B*, etc.), suggesting a broader contribution of these genes to ID and disease than previously reported. This emphasizes the need for non‐biased methods for diagnosis. Previous studies have observed similar phenotypic variability in individuals with mutations in known syndromic ID‐associated genes (e.g., *ARID1B*, *RPS6KA3* [MIM #300075], *TCF4, ATRX, SATB2* [MIM #608148] and *SCN8A* [MIM #600702]) [Guerrini et al., 2000; Field et al., 2006; Hoyer et al., 2012; Rauch et al., 2012; Santen et al., 2012; Santen et al., 2013]. Initial studies implicating a gene in disease tend to be enriched for the most phenotypically extreme cases based on clinical ascertainment rather than a cohort representative of the population. This may explain how the initial estimates of penetrance tend to be inflated, whereas the phenotypic heterogeneity tends to be underestimated [Hoischen et al., 2014]. Therefore, large sample sets of ID will provide more data to estimate the full scope of the associated phenotype presentation of mutations in a particular gene.

The second aim of this study was to attempt to identify any novel ID‐associated genes, within those genes currently classified as candidate genes. In this study, the CAST test was underpowered to detect a significant enrichment of variants in individual genes. This would require much larger sample sizes, as has also been shown by other studies [Liu et al., 2013]. Nevertheless, the finding of an enrichment of LoF variants in candidate ID‐associated genes shows that some of these variants are probably causative. We found additional evidence in support of *ZMYM6, PHF10, PHIP, ASH1L, WAC* being novel autosomal‐dominant ID genes, and *FAM120C*, *WNK3* novel X‐linked disease genes and report further rare variants in *TRMT1*, an autosomal‐recessive gene but the cohort alone did not provide sufficient evidence to definitively reclassify these candidate ID‐associated genes to known gene status. Only *SETD5*, which we previously reported, yielded sufficient number of rare LoF variants to identify a novel gene from within the cohort alone [Grozeva et al., 2014]. Assigning causality to a novel candidate gene requires a high degree of evidence and further sharing of data from similarly large cohorts of cases with ID is needed to identify the remaining rarer novel disease causing genes for this highly heterogeneous disorder [MacArthur et al., 2014].

A recent study of Redin et al. (2014) also argued the benefits of using targeted NGS approach in a large cohort as a first intention test for diagnosis of ID. It was shown that variants in some genes were more frequently observed than others (*MECP2*, *CUL4B*, *IL1RAPL1*, *TCF4*, *SLC2A1*, *KDM5C* [MIM #314690], etc.). Therefore, the authors concluded that introducing the sequencing of these genes in clinical practice could be a useful analysis to perform [Redin et al., 2014]. While our data support the above list, we can add more genes: *SETD5*, *ARID1B*, and *ATRX*. Furthermore, it has been shown that genes involved in developmental disorders have low‐intolerance scores and thus damaging variants in these genes are not likely to occur frequently in the general population [Petrovski et al., 2013]. This could justify sequencing these genes as part of a diagnostic panel when parents could not be recruited for de novo analysis.

The overall diagnostic rate was ∼11% of the cohort. This yield is somewhat lower than observed in trio‐based ID studies, where yields ranged from 13% to 28% in exome sequencing analysis and 42% in a recent whole‐genome analysis [de Ligt et al., 2012; Rauch et al., 2012; Gilissen et al., 2014; Hamdan et al., 2014; The Deciphering Developmental Disorders Study, 2014]. There are notable differences in inclusion criteria and methodology between our study and the studies above, which make direct comparisons of diagnostic yield between the studies problematic. However, the diagnostic yield of ∼11% in our study indicates significant clinical utility of a proband‐only approach where one or both parents are unavailable for testing. For the identification of novel ID‐associated genes, trio analysis remains the strategy of choice, although the power to detect additional variants in candidate genes in a replication cohort is illustrated here.

In conclusion, our study shows that targeted NGS of a limited number of ID‐associated genes in probands with moderate‐to‐severe ID is a valuable approach and could provide a diagnosis in ∼11% of the individuals. This rate is comparable to the diagnostic rate of CGH microarray analysis (∼14% for copy‐number variants >400kb) [Cooper et al., 2011]. Such analysis could supplement the currently first tier copy‐number microarray test in providing genetic diagnosis for families where DNA samples from both parents are not available for de novo analysis.

*Disclosure statement*: The authors declare no conflict of interest.

## Supporting information


**Figure S1**. Variants per person. All variants with MAF<1% taken into account. N‐ number
**Figure S2**. LoF variants per person. Samples with >30 variants were excluded. N‐ number
**Figure S3**. Missense variants per person. Samples with >30 variants were excluded. N‐ number
**Figure S4**. Principal component analysis. The first two eigenvectors (EVs) cluster the Hapmap3.3 samples into their component populations (AFR = individuals of African ancestry; ASN = individuals of East Asian ancestry; SAN = individuals of South Asian ancestry; EUR = individuals of European ancestry) [Altshuler et al., 2010]. There is no qualitative difference in population structure between the ID and CHD cohorts
**Table S1**. List sequenced genes
**Table S3**. Likely causative LoF variants
**Table S4**. Likely causative missense variants

**Table S2**. All rare, coding SNPs and indels identified in this study, including non‐pathogenic variants
