# Supplementary material for: Targeted Next‐Generation Sequencing Analysis of 1,000 Individuals with Intellectual Disability
Source: Hum Mutat. 2015 Sep 30;36(12):1197–204. doi: 10.1002/humu.22901 (PMC4833192; doi:10.1002/humu.22901)
Supplement: Supplementary file 1 — Figure S1. Variants per person. All variants with MAF<1% taken into account. N‐ number Figure S2. LoF variants per person. Samples with >30 variants were excluded. N‐ number Figure S3. Missense variants per person. Samples with >30 variants were excluded. N‐ number Figure S4. Principal component analysis. The first two eigenvectors (EVs) cluster the Hapmap3.3 samples into their component populations (AFR = individuals of African ancestry; ASN = individuals of East Asian ancestry; SAN = individuals of South Asian ancestry; EUR = individuals of European ancestry) [Altshuler et al., 2010]. There is no qualitative difference in population structure between the ID and CHD cohorts Table S1. List sequenced genes Table S3. Likely causative LoF variants Table S4. Likely causative missense variants [file HUMU-36-1197-s001.pdf]

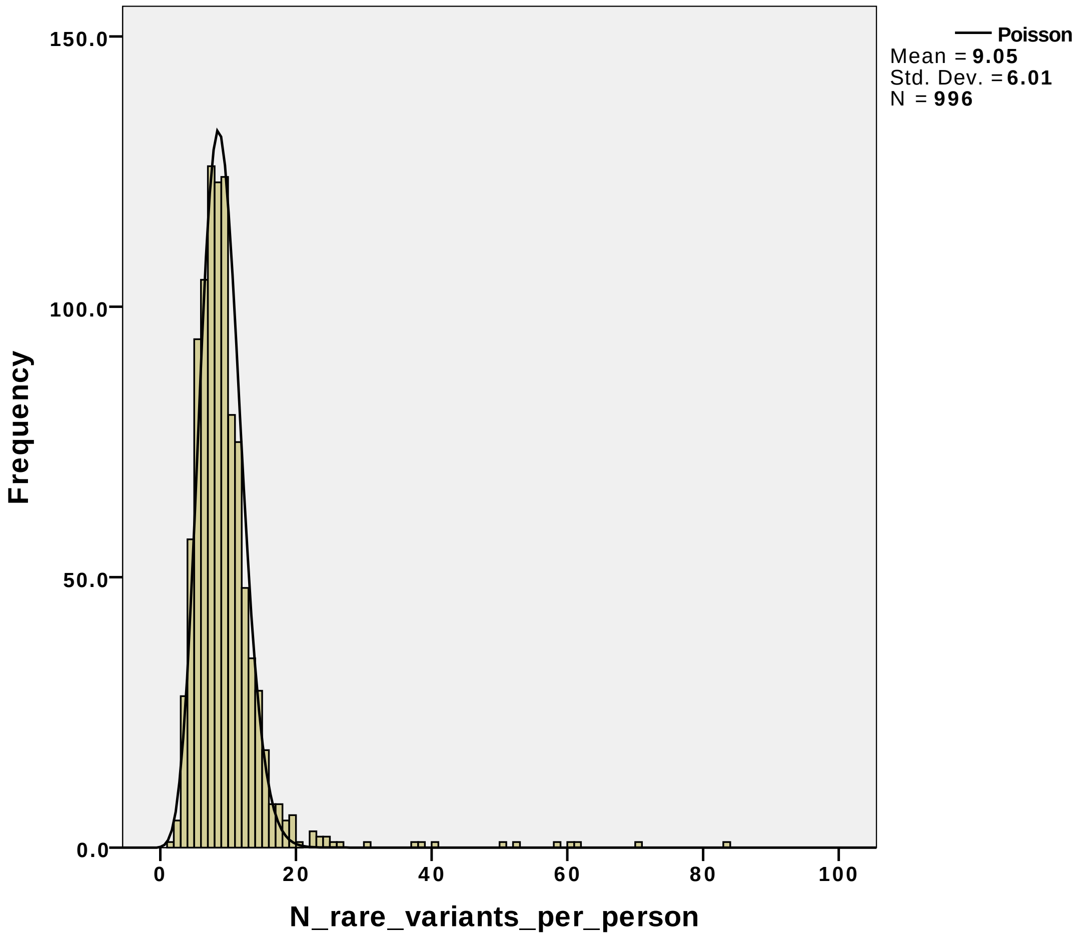

Supp. Figure S1. Variants per person. All variants with MAF<1% taken into account.
N- number

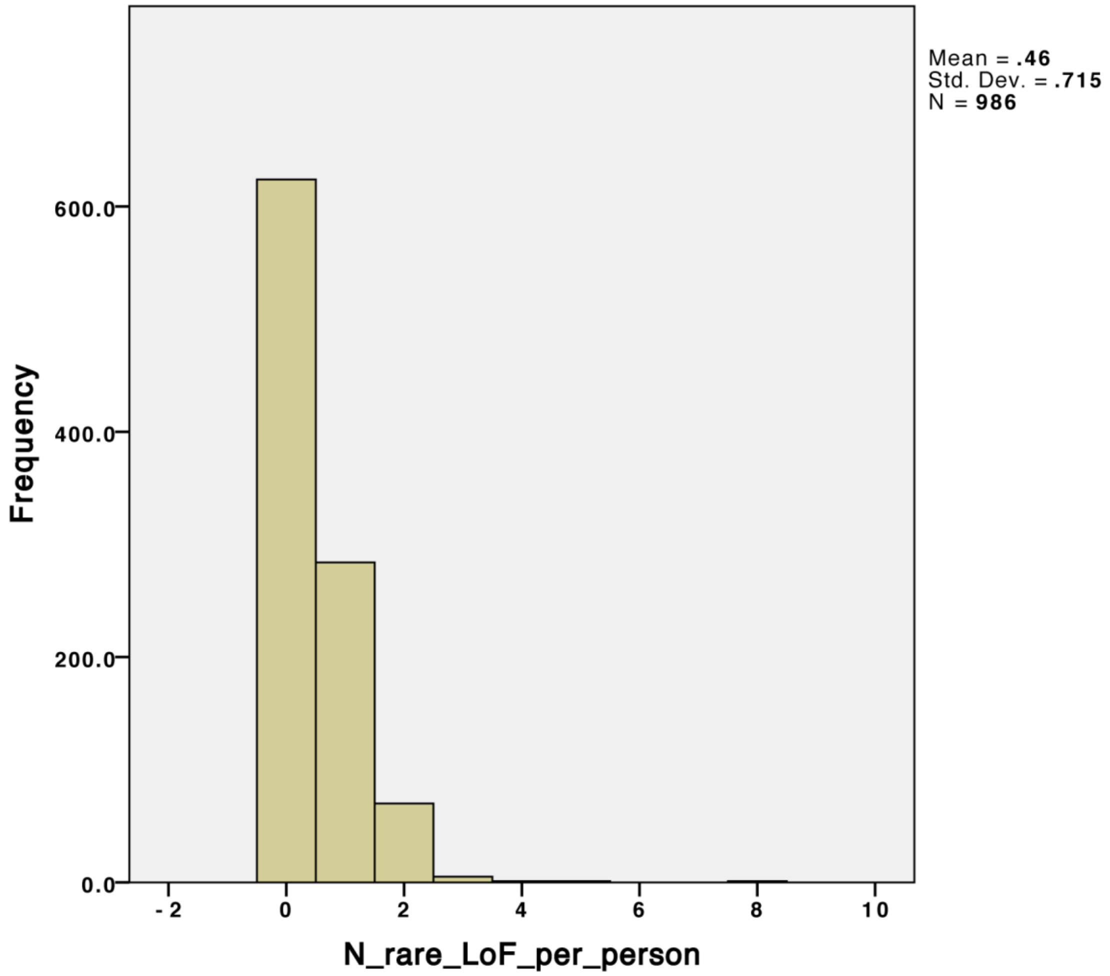

Supp. Figure S2. LoF variants per person. Samples with >30 variants were excluded.
N- number

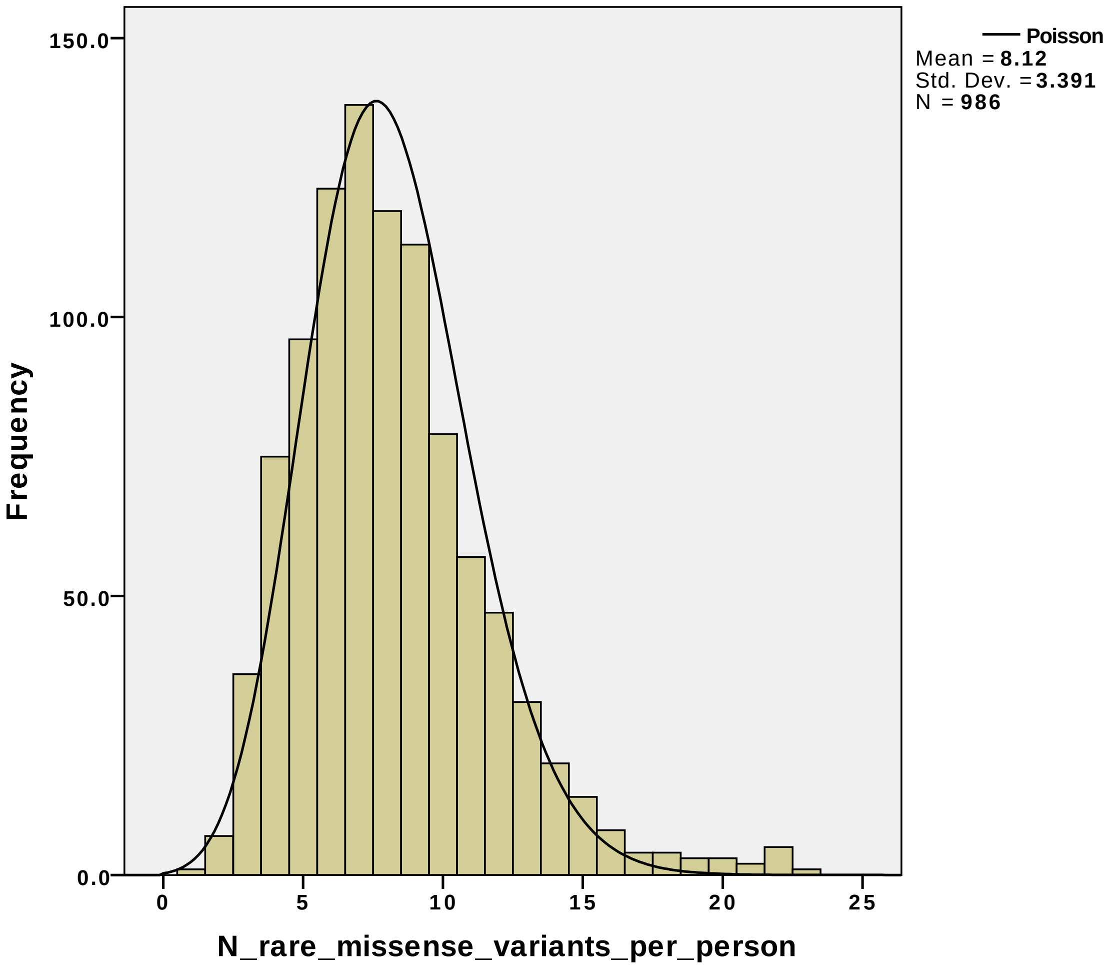

Supp. Figure S3. Missense variants per person. Samples with >30 variants were excluded. N- number

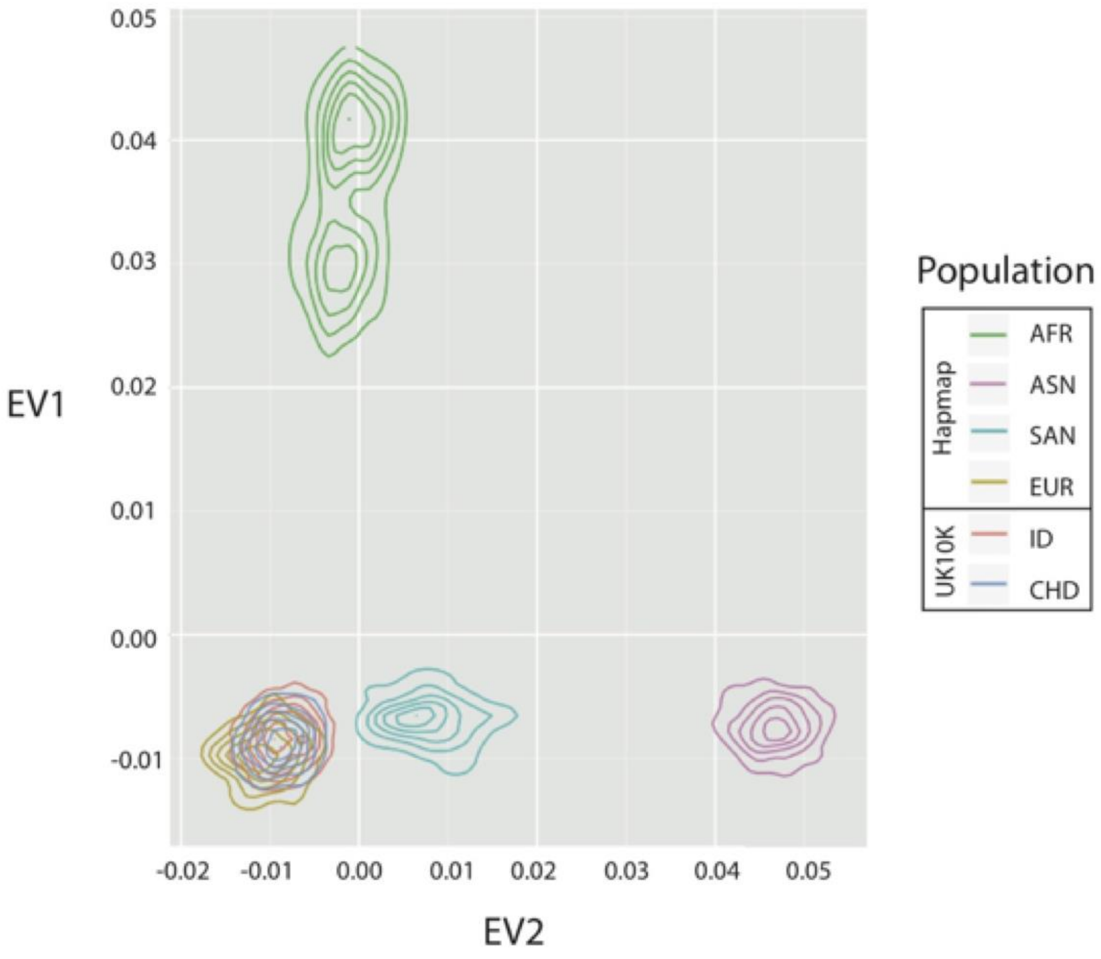

Supp. Figure S4. Principal component analysis. The first two eigenvectors (EVs) cluster the Hapmap3.3 samples into their component populations (AFR = individuals of African ancestry; ASN = individuals of East Asian ancestry; SAN = individuals of South Asian ancestry; EUR = individuals of European ancestry) [Altshuler et al., 2010]. There is no qualitative difference in population structure between the ID and CHD cohorts.

Supp. Table S1. List sequenced genes

Chromosome	Gene ID	Known or a Candidate Gene	Origin
1	*AP4B1*	Known	in-house curation
2	*KIF5C*	Known	in-house curation
3	*SETD5*	Known	in-house curation
9	*MAN1B1*	Known	in-house curation
14	*AP4S1*	Known	in-house curation
15	*AP4E1*	Known	in-house curation
X	*MAOA*	Known	in-house curation
X	*ARHGEF6*	Known	in-house curation
X	*ACSL4*	Known	in-house curation
X	*USP9X*	Known	in-house curation
1	*PARP1*	Known	Gilissen et al. 2014
1	*SLC26A9*	Known	Gilissen et al. 2014
2	*MBD5*	Known	Gilissen et al. 2014
2	*MYT1L*	Known	Gilissen et al. 2014
2	*NRXN1*	Known	Gilissen et al. 2014
2	*MLL3*	Known	Gilissen et al. 2014
3	*SRGAP3*	Known	Gilissen et al. 2014
3	*MLH1*	Known	Gilissen et al. 2014
5	*NSUN2*	Known	Gilissen et al. 2014
8	*KCNQ3*	Known	Gilissen et al. 2014
8	*ZFHX4*	Known	Gilissen et al. 2014
9	*KANK1*	Known	Gilissen et al. 2014
9	*SPTAN1*	Known	Gilissen et al. 2014
10	*ANK3*	Known	Gilissen et al. 2014
11	*SHANK2*	Known	Gilissen et al. 2014
12	*LRP1*	Known	Gilissen et al. 2014
12	*SOX5*	Known	Gilissen et al. 2014
14	*DYNC1H1*	Known	Gilissen et al. 2014
16	*TBC1D24*	Known	Gilissen et al. 2014
17	*SMARCE1*	Known	Gilissen et al. 2014
19	*SMARCA4*	Known	Gilissen et al. 2014
20	*ARFGEF2*	Known	Gilissen et al. 2014
22	*SHANK3*	Known	Gilissen et al. 2014
X	*NLGN4X*	Known	Gilissen et al. 2014
X	*AGTR2*	Known	Gilissen et al. 2014
X	*ARHGEF9*	Known	Gilissen et al. 2014
X	*SHROOM4*	Known	Gilissen et al. 2014
X	*ZNF41*	Known	Gilissen et al. 2014
X	*ZNF674*	Known	Gilissen et al. 2014
X	*SMS*	Known	Gilissen et al. 2014
X	*CCDC22*	Known	Gilissen et al. 2014
X	*SYN1*	Known	Gilissen et al. 2014
1	*SLC2A1*	Known	DDG2P list, Gilissen et al. 2014
1	*ADCK3*	Known	DDG2P list, Gilissen et al. 2014
1	*ALG6*	Known	DDG2P list, Gilissen et al. 2014
1	*ARID1A*	Known	DDG2P list, Gilissen et al. 2014
1	*GJC2*	Known	DDG2P list, Gilissen et al. 2014
2	*SATB2*	Known	DDG2P list, Gilissen et al. 2014
2	*SCN2A*	Known	DDG2P list, Gilissen et al. 2014
2	*HDAC4*	Known	DDG2P list, Gilissen et al. 2014
2	*LRP2*	Known	DDG2P list, Gilissen et al. 2014

Chromosome	Gene ID	Known or a Candidate Gene	Origin
2	*MMADHC*	Known	DDG2P list, Gilissen et al. 2014
2	*RAB3GAP1*	Known	DDG2P list, Gilissen et al. 2014
2	*SOS1*	Known	DDG2P list, Gilissen et al. 2014
2	*ZEB2*	Known	DDG2P list, Gilissen et al. 2014
3	*FOXP1*	Known	DDG2P list, Gilissen et al. 2014
3	*RAF1*	Known	DDG2P list, Gilissen et al. 2014
3	*CTNNB1*	Known	DDG2P list, Gilissen et al. 2014
3	*ALG3*	Known	DDG2P list, Gilissen et al. 2014
4	*AGA*	Known	DDG2P list, Gilissen et al. 2014
4	*CC2D2A*	Known	DDG2P list, Gilissen et al. 2014
4	*IDUA*	Known	DDG2P list, Gilissen et al. 2014
4	*MMAA*	Known	DDG2P list, Gilissen et al. 2014
5	*MEF2C*	Known	DDG2P list, Gilissen et al. 2014
5	*NSD1*	Known	DDG2P list, Gilissen et al. 2014
6	*SYNE1*	Known	DDG2P list, Gilissen et al. 2014
6	*ARID1B*	Known	DDG2P list, Gilissen et al. 2014
6	*ALDH5A1*	Known	DDG2P list, Gilissen et al. 2014
6	*NEU1*	Known	DDG2P list, Gilissen et al. 2014
6	*SYNGAP1*	Known	DDG2P list, Gilissen et al. 2014
6	*TUBB2B*	Known	DDG2P list, Gilissen et al. 2014
7	*CNTNAP2*	Known	DDG2P list, Gilissen et al. 2014
7	*BRAF*	Known	DDG2P list, Gilissen et al. 2014
7	*CEP41*	Known	DDG2P list, Gilissen et al. 2014
8	*CHD7*	Known	DDG2P list, Gilissen et al. 2014
8	*TRAPPC9*	Known	DDG2P list, Gilissen et al. 2014
8	*TUSC3*	Known	DDG2P list, Gilissen et al. 2014
8	*VPS13B*	Known	DDG2P list, Gilissen et al. 2014
9	*EHMT1*	Known	DDG2P list, Gilissen et al. 2014
9	*AUH*	Known	DDG2P list, Gilissen et al. 2014
9	*EXOSC3*	Known	DDG2P list, Gilissen et al. 2014
9	*FKTN*	Known	DDG2P list, Gilissen et al. 2014
9	*INPP5E*	Known	DDG2P list, Gilissen et al. 2014
9	*SMARCA2*	Known	DDG2P list, Gilissen et al. 2014
9	*STXBP1*	Known	DDG2P list, Gilissen et al. 2014
9	*TSC1*	Known	DDG2P list, Gilissen et al. 2014
9	*VLDLR*	Known	DDG2P list, Gilissen et al. 2014
10	*PTEN*	Known	DDG2P list, Gilissen et al. 2014
10	*KAT6B*	Known	DDG2P list, Gilissen et al. 2014
10	*ALDH18A1*	Known	DDG2P list, Gilissen et al. 2014
10	*ERCC6*	Known	DDG2P list, Gilissen et al. 2014
10	*POLR3A*	Known	DDG2P list, Gilissen et al. 2014
10	*SHOC2*	Known	DDG2P list, Gilissen et al. 2014
10	*WDR11*	Known	DDG2P list, Gilissen et al. 2014
11	*PAX6*	Known	DDG2P list, Gilissen et al. 2014
11	*MLL2*	Known	DDG2P list, Gilissen et al. 2014
11	*DHCR7*	Known	DDG2P list, Gilissen et al. 2014
11	*HRAS*	Known	DDG2P list, Gilissen et al. 2014
11	*KIRREL3*	Known	DDG2P list, Gilissen et al. 2014
11	*PC*	Known	DDG2P list, Gilissen et al. 2014
12	*SCN8A*	Known	DDG2P list, Gilissen et al. 2014
12	*GRIN2B*	Known	DDG2P list, Gilissen et al. 2014
12	*KRAS*	Known	DDG2P list, Gilissen et al. 2014
12	*POLR3B*	Known	DDG2P list, Gilissen et al. 2014

Chromosome	Gene ID	Known or a Candidate Gene	Origin
12	*PTPN11*	Known	DDG2P list, Gilissen et al. 2014
12	*TUBA1A*	Known	DDG2P list, Gilissen et al. 2014
14	*FOXG1*	Known	DDG2P list, Gilissen et al. 2014
14	*GCH1*	Known	DDG2P list, Gilissen et al. 2014
14	*ZFYVE26*	Known	DDG2P list, Gilissen et al. 2014
15	*MAP2K1*	Known	DDG2P list, Gilissen et al. 2014
15	*UBE3A*	Known	DDG2P list, Gilissen et al. 2014
15	*CHD2*	Known	DDG2P list, Gilissen et al. 2014
15	*KIF7*	Known	DDG2P list, Gilissen et al. 2014
15	*SLC12A6*	Known	DDG2P list, Gilissen et al. 2014
15	*SPRED1*	Known	DDG2P list, Gilissen et al. 2014
15	*UBR1*	Known	DDG2P list, Gilissen et al. 2014
16	*CREBBP*	Known	DDG2P list, Gilissen et al. 2014
16	*ALG1*	Known	DDG2P list, Gilissen et al. 2014
16	*CDH15*	Known	DDG2P list, Gilissen et al. 2014
16	*GPR56*	Known	DDG2P list, Gilissen et al. 2014
16	*GRIN2A*	Known	DDG2P list, Gilissen et al. 2014
16	*MLYCD*	Known	DDG2P list, Gilissen et al. 2014
16	*NDE1*	Known	DDG2P list, Gilissen et al. 2014
16	*TAT*	Known	DDG2P list, Gilissen et al. 2014
16	*TSC2*	Known	DDG2P list, Gilissen et al. 2014
17	*KANSL1*	Known	DDG2P list, Gilissen et al. 2014
17	*NF1*	Known	DDG2P list, Gilissen et al. 2014
17	*PAFAH1B1*	Known	DDG2P list, Gilissen et al. 2014
17	*RAI1*	Known	DDG2P list, Gilissen et al. 2014
18	*SETBP1*	Known	DDG2P list, Gilissen et al. 2014
18	*TCF4*	Known	DDG2P list, Gilissen et al. 2014
19	*FKRP*	Known	DDG2P list, Gilissen et al. 2014
19	*MAP2K2*	Known	DDG2P list, Gilissen et al. 2014
19	*NFIX*	Known	DDG2P list, Gilissen et al. 2014
19	*PEPD*	Known	DDG2P list, Gilissen et al. 2014
19	*PNKP*	Known	DDG2P list, Gilissen et al. 2014
19	*WDR62*	Known	DDG2P list, Gilissen et al. 2014
20	*ASXL1*	Known	DDG2P list, Gilissen et al. 2014
20	*DNMT3B*	Known	DDG2P list, Gilissen et al. 2014
21	*DYRK1A*	Known	DDG2P list, Gilissen et al. 2014
21	*PCNT*	Known	DDG2P list, Gilissen et al. 2014
22	*ADSL*	Known	DDG2P list, Gilissen et al. 2014
22	*ALG12*	Known	DDG2P list, Gilissen et al. 2014
22	*EP300*	Known	DDG2P list, Gilissen et al. 2014
22	*SMARCB1*	Known	DDG2P list, Gilissen et al. 2014
X	*ABCD1*	Known	DDG2P list, Gilissen et al. 2014
X	*FMR1*	Known	DDG2P list, Gilissen et al. 2014
X	*GK*	Known	DDG2P list, Gilissen et al. 2014
X	*TIMM8A*	Known	DDG2P list, Gilissen et al. 2014
X	*AFF2*	Known	DDG2P list, Gilissen et al. 2014
X	*GPC3*	Known	DDG2P list, Gilissen et al. 2014
X	*KDM5C*	Known	DDG2P list, Gilissen et al. 2014
X	*OFD1*	Known	DDG2P list, Gilissen et al. 2014
X	*PTCHD1*	Known	DDG2P list, Gilissen et al. 2014
X	*SMC1A*	Known	DDG2P list, Gilissen et al. 2014
X	*TSPAN7*	Known	DDG2P list, Gilissen et al. 2014
X	*OPHN1*	Known	DDG2P list, Gilissen et al. 2014

Chromosome	Gene ID	Known or a Candidate Gene	Origin
X	*SLC9A6*	Known	DDG2P list, Gilissen et al. 2014
X	*UPF3B*	Known	DDG2P list, Gilissen et al. 2014
X	*ZDHHC9*	Known	DDG2P list, Gilissen et al. 2014
X	*IL1RAPL1*	Known	DDG2P list, Gilissen et al. 2014
X	*ATRX*	Known	DDG2P list, Gilissen et al. 2014
X	*CUL4B*	Known	DDG2P list, Gilissen et al. 2014
X	*AP1S2*	Known	DDG2P list, Gilissen et al. 2014
X	*ARX*	Known	DDG2P list, Gilissen et al. 2014
X	*ATP7A*	Known	DDG2P list, Gilissen et al. 2014
X	*BCOR*	Known	DDG2P list, Gilissen et al. 2014
X	*CASK*	Known	DDG2P list, Gilissen et al. 2014
X	*CDKL5*	Known	DDG2P list, Gilissen et al. 2014
X	*DCX*	Known	DDG2P list, Gilissen et al. 2014
X	*DKC1*	Known	DDG2P list, Gilissen et al. 2014
X	*DLG3*	Known	DDG2P list, Gilissen et al. 2014
X	*DMD*	Known	DDG2P list, Gilissen et al. 2014
X	*FGD1*	Known	DDG2P list, Gilissen et al. 2014
X	*FLNA*	Known	DDG2P list, Gilissen et al. 2014
X	*FTSJ1*	Known	DDG2P list, Gilissen et al. 2014
X	*GDI1*	Known	DDG2P list, Gilissen et al. 2014
X	*GRIA3*	Known	DDG2P list, Gilissen et al. 2014
X	*HCCS*	Known	DDG2P list, Gilissen et al. 2014
X	*HCFC1*	Known	DDG2P list, Gilissen et al. 2014
X	*HDAC8*	Known	DDG2P list, Gilissen et al. 2014
X	*HPRT1*	Known	DDG2P list, Gilissen et al. 2014
X	*HSD17B10*	Known	DDG2P list, Gilissen et al. 2014
X	*HUWE1*	Known	DDG2P list, Gilissen et al. 2014
X	*IDS*	Known	DDG2P list, Gilissen et al. 2014
X	*IKBKG*	Known	DDG2P list, Gilissen et al. 2014
X	*IQSEC2*	Known	DDG2P list, Gilissen et al. 2014
X	*L1CAM*	Known	DDG2P list, Gilissen et al. 2014
X	*LAMP2*	Known	DDG2P list, Gilissen et al. 2014
X	*MECP2*	Known	DDG2P list, Gilissen et al. 2014
X	*MED12*	Known	DDG2P list, Gilissen et al. 2014
X	*MID1*	Known	DDG2P list, Gilissen et al. 2014
X	*NDP*	Known	DDG2P list, Gilissen et al. 2014
X	*NHS*	Known	DDG2P list, Gilissen et al. 2014
X	*NSDHL*	Known	DDG2P list, Gilissen et al. 2014
X	*OCRL*	Known	DDG2P list, Gilissen et al. 2014
X	*OTC*	Known	DDG2P list, Gilissen et al. 2014
X	*PAK3*	Known	DDG2P list, Gilissen et al. 2014
X	*PCDH19*	Known	DDG2P list, Gilissen et al. 2014
X	*PDHA1*	Known	DDG2P list, Gilissen et al. 2014
X	*PGK1*	Known	DDG2P list, Gilissen et al. 2014
X	*PHF6*	Known	DDG2P list, Gilissen et al. 2014
X	*PHF8*	Known	DDG2P list, Gilissen et al. 2014
X	*PLP1*	Known	DDG2P list, Gilissen et al. 2014
X	*PORCN*	Known	DDG2P list, Gilissen et al. 2014
X	*PRPS1*	Known	DDG2P list, Gilissen et al. 2014
X	*RPS6KA3*	Known	DDG2P list, Gilissen et al. 2014
X	*SHOX*	Known	DDG2P list, Gilissen et al. 2014
X	*SLC16A2*	Known	DDG2P list, Gilissen et al. 2014
X	*SLC6A8*	Known	DDG2P list, Gilissen et al. 2014

Chromosome	Gene ID	Known or a Candidate Gene	Origin
X	*SOX3*	Known	DDG2P list, Gilissen et al. 2014
X	*SYP*	Known	DDG2P list, Gilissen et al. 2014
X	*UBE2A*	Known	DDG2P list, Gilissen et al. 2014
1	*ALDH4A1*	Known	DDG2P list
1	*DDOST*	Known	DDG2P list
1	*PPT1*	Known	DDG2P list
2	*KIF1A*	Known	DDG2P list
3	*ACY1*	Known	DDG2P list
3	*GLB1*	Known	DDG2P list
4	*PRSS12*	Known	DDG2P list
5	*GM2A*	Known	DDG2P list
5	*HEXB*	Known	DDG2P list
5	*OXCT1*	Known	DDG2P list
6	*GRIK2*	Known	DDG2P list
6	*ARG1*	Known	DDG2P list
6	*RNASET2*	Known	DDG2P list
8	*CA8*	Known	DDG2P list
8	*HGSNAT*	Known	DDG2P list
11	*DEAF1*	Known	DDG2P list
11	*ALG8*	Known	DDG2P list
11	*ATM*	Known	DDG2P list
11	*CTSD*	Known	DDG2P list
12	*PAH*	Known	DDG2P list
12	*MMAB*	Known	DDG2P list
15	*HEXA*	Known	DDG2P list
15	*SPG11*	Known	DDG2P list
16	*PRRT2*	Known	DDG2P list
17	*COX10*	Known	DDG2P list
17	*SGSH*	Known	DDG2P list
17	*TSEN54*	Known	DDG2P list
19	*CC2D1A*	Known	DDG2P list
19	*FTL*	Known	DDG2P list
19	*GCDH*	Known	DDG2P list
22	*MLC1*	Known	DDG2P list
22	*TUBA8*	Known	DDG2P list
X	*BRWD3*	Known	DDG2P list
X	*PQBP1*	Known	DDG2P list
X	*NDUFA1*	Known	DDG2P list
X	*RAB39B*	Known	DDG2P list
X	*ZNF711*	Known	DDG2P list
X	*FAM58A*	Known	DDG2P list
X	*IGSF1*	Known	DDG2P list
X	*TM4SF2*	Known	DDG2P list
1	*ASH1L*	candidate gene	
1	*ZMYM6*	candidate gene	
1	*ACBD6*	candidate gene	
1	*CNKSR1*	candidate gene	
1	*GATAD2B*	candidate gene	
1	*GON4L*	candidate gene	
1	*HIST3H3*	candidate gene	
1	*MTF1*	candidate gene	
1	*RGS7*	candidate gene	
1	*ZBTB40*	candidate gene	

Chromosome	Gene ID	Known or a Candidate Gene	Origin
1	*ST3GAL3*	candidate gene	
1	*KDM1A*	candidate gene	
1	*CAP1*	candidate gene	
1	*KCNH1*	candidate gene	
1	*ZNF238*	candidate gene	
1	*NR1I3*	candidate gene	
1	*BDP1*	candidate gene	
1	*CCDC23*	candidate gene	
1	*KIF26B*	candidate gene	
1	*KLHL21*	candidate gene	
1	*ODF2L*	candidate gene	
1	*SLC6A17*	candidate gene	
1	*ZMYND12*	candidate gene	
2	*ADRA2B*	candidate gene	
2	*EEF1B2*	candidate gene	
2	*GAD1*	candidate gene	
2	*PECR*	candidate gene	
2	*GRB14*	candidate gene	
2	*KCNK12*	candidate gene	
2	*HSPD1*	candidate gene	
2	*CAPN10*	candidate gene	
2	*INPP4A*	candidate gene	
2	*ARHGEF4*	candidate gene	
2	*ITGA4*	candidate gene	
3	*CHL1*	candidate gene	
3	*DLG1*	candidate gene	
3	*GTPBP8*	candidate gene	
3	*PBRM1*	candidate gene	
3	*SLC6A1*	candidate gene	
3	*STAG1*	candidate gene	
3	*TSEN2*	candidate gene	
3	*ACTL6A*	candidate gene	
3	*DHX30*	candidate gene	
3	*SMARCC1*	candidate gene	
4	*LARP7*	candidate gene	
4	*PRMT10*	candidate gene	
4	*AIMP1*	candidate gene	
4	*GRIA2*	candidate gene	
4	*CCNA2*	candidate gene	
4	*DCHS2*	candidate gene	
4	*PCDH10*	candidate gene	
5	*NDST1*	candidate gene	
5	*TRIO*	candidate gene	
5	*COL4A3BP*	candidate gene	
5	*GRIA1*	candidate gene	
5	*CAMK2A*	candidate gene	
6	*PHIP*	candidate gene	
6	*ASCC3*	candidate gene	
6	*MED23*	candidate gene	
6	*HIVEP2*	candidate gene	
6	*PHACTR1*	candidate gene	
6	*PPP2R5D*	candidate gene	
6	*SYNCRIP*	candidate gene	

Chromosome	Gene ID	Known or a Candidate Gene	Origin
6	*HIST1H4B*	candidate gene	
6	*PHF10*	candidate gene	
6	*FKBPL*	candidate gene	
6	*TCP10L2*	candidate gene	
6	*TENM1*	candidate gene	
7	*CASP2*	candidate gene	
7	*CTTNBP2*	candidate gene	
7	*AP4M1*	candidate gene	
7	*ACTL6B*	candidate gene	
7	*LHFPL3*	candidate gene	
7	*LIMK1*	candidate gene	
7	*MYO1G*	candidate gene	
7	*SMARCD3*	candidate gene	
7	*ZNF425*	candidate gene	
8	*ERLIN2*	candidate gene	
8	*TAF2*	candidate gene	
8	*TTI2*	candidate gene	
8	*THAP1*	candidate gene	
8	*CYP7B1*	candidate gene	
8	*EPPK1*	candidate gene	
8	*SNTG1*	candidate gene	
9	*RALGDS*	candidate gene	
9	*SLC31A1*	candidate gene	
9	*RAPGEF1*	candidate gene	
9	*RABL6*	candidate gene	
10	*WAC*	candidate gene	
10	*ADK*	candidate gene	
10	*ENTPD1*	candidate gene	
10	*CAMK2G*	candidate gene	
10	*TNKS2*	candidate gene	
10	*TUBAL3*	candidate gene	
11	*TMEM135*	candidate gene	
11	*MED17*	candidate gene	
11	*SLC25A22*	candidate gene	
11	*OR5M1*	candidate gene	
11	*ARL14EP*	candidate gene	
11	*NRXN2*	candidate gene	
11	*DLG2*	candidate gene	
11	*DPF2*	candidate gene	
11	*NTM*	candidate gene	
12	*ASCL1*	candidate gene	
12	*C12orf57*	candidate gene	
12	*COQ5*	candidate gene	
12	*KDM5A*	candidate gene	
12	*ZCCHC8*	candidate gene	
12	*CUX2*	candidate gene	
12	*SMARCC2*	candidate gene	
12	*ARID2*	candidate gene	
12	*SMARCD1*	candidate gene	
12	*STAB2*	candidate gene	
12	*SYT1*	candidate gene	
13	*FRY*	candidate gene	
13	*DGKH*	candidate gene	

Chromosome	Gene ID	Known or a Candidate Gene	Origin
13	*CDK8*	candidate gene	
13	*SETDB2*	candidate gene	
14	*UBR7*	candidate gene	
14	*YY1*	candidate gene	
14	*PROX2*	candidate gene	
14	*ZC3H14*	candidate gene	
14	*SPTLC2*	candidate gene	
14	*VRK1*	candidate gene	
14	*ACIN1*	candidate gene	
14	*DPF3*	candidate gene	
14	*PTPN21*	candidate gene	
15	*LINS*	candidate gene	
15	*ARIH1*	candidate gene	
15	*SCAPER*	candidate gene	
15	*LRRK1*	candidate gene	
16	*NECAB2*	candidate gene	
16	*THUMPD1*	candidate gene	
17	*CACNA1G*	candidate gene	
17	*FASN*	candidate gene	
17	*KDM6B*	candidate gene	
17	*DLG4*	candidate gene	
17	*TANC2*	candidate gene	
17	*UBTF*	candidate gene	
17	*WDR45L*	candidate gene	
17	*ENTHD2*	candidate gene	
17	*MGAT5B*	candidate gene	
17	*MYO1D*	candidate gene	
17	*SMARCD2*	candidate gene	
17	*TMEM132E*	candidate gene	
18	*ELP2*	candidate gene	
18	*LAMA1*	candidate gene	
18	*PIGN*	candidate gene	
18	*MIB1*	candidate gene	
18	*PIK3C3*	candidate gene	
19	*TRMT1*	candidate gene	
19	*ZNF526*	candidate gene	
19	*TNPO2*	candidate gene	
19	*KCNC3*	candidate gene	
19	*SHANK1*	candidate gene	
19	*TSEN34*	candidate gene	
19	*DPF1*	candidate gene	
20	*EEF1A2*	candidate gene	
20	*PSMA7*	candidate gene	
21	*IFNAR2*	candidate gene	
22	*PLA2G6*	candidate gene	
22	*SREBF2*	candidate gene	
X	*KIAA2022*	candidate gene	
X	*MAGT1*	candidate gene	
X	*NXF5*	candidate gene	
X	*ZDHHC15*	candidate gene	
X	*ZMYM3*	candidate gene	
X	*GSPT2*	candidate gene	
X	*NAA10*	candidate gene	

Chromosome	Gene ID	Known or a Candidate Gene	Origin
X	*RBM10*	candidate gene	
X	*SRPX2*	candidate gene	
X	*BCORL1*	candidate gene	
X	*CLIC2*	candidate gene	
X	*CNKSR2*	candidate gene	
X	*DDX3X*	candidate gene	
X	*EIF2C1*	candidate gene	
X	*EIF2S3*	candidate gene	
X	*ELK1*	candidate gene	
X	*FAAH2*	candidate gene	
X	*FRMPD4*	candidate gene	
X	*PGRMC1*	candidate gene	
X	*RAB40AL*	candidate gene	
X	*TAF1*	candidate gene	
X	*TAF7L*	candidate gene	
X	*THOC2*	candidate gene	
X	*TMLHE*	candidate gene	
X	*WDR13*	candidate gene	
X	*ZCCHC12*	candidate gene	
X	*ZNF81*	candidate gene	
X	*ALG13*	candidate gene	
X	*MAOB*	candidate gene	
X	*NLGN3*	candidate gene	
X	*FAM120C*	candidate gene	
X	*SYTL5*	candidate gene	
X	*WNK3*	candidate gene	
X	*ACE2*	candidate gene	
X	*ACOT9*	candidate gene	
X	*AKAP17A*	candidate gene	
X	*AKAP4*	candidate gene	
X	*ARHGAP36*	candidate gene	
X	*ARHGAP6*	candidate gene	
X	*ARSF*	candidate gene	
X	*ASB12*	candidate gene	
X	*ASMT*	candidate gene	
X	*ASMTL*	candidate gene	
X	*ATP2B3*	candidate gene	
X	*ATXN3L*	candidate gene	
X	*AVPR2*	candidate gene	
X	*AWAT2*	candidate gene	
X	*BMP15*	candidate gene	
X	*BTK*	candidate gene	
X	*CACNA1F*	candidate gene	
X	*CCNB3*	candidate gene	
X	*CD99*	candidate gene	
X	*CDK16*	candidate gene	
X	*CFP*	candidate gene	
X	*CLCN4*	candidate gene	
X	*CLCN5*	candidate gene	
X	*CMC4*	candidate gene	
X	*COL4A6*	candidate gene	
X	*CPXCR1*	candidate gene	
X	*CRLF2*	candidate gene	

Chromosome	Gene ID	Known or a Candidate Gene	Origin
X	*CSF2RA*	candidate gene	
X	*CSTF2*	candidate gene	
X	*CTPS2*	candidate gene	
X	*CXORF22*	candidate gene	
X	*CXORF58*	candidate gene	
X	*DDX26B*	candidate gene	
X	*DDX53*	candidate gene	
X	*DHRSX*	candidate gene	
X	*DIAPH2*	candidate gene	
X	*DOCK11*	candidate gene	
X	*ENOX2*	candidate gene	
X	*ESX1*	candidate gene	
X	*FAM47B*	candidate gene	
X	*GAB3*	candidate gene	
X	*GABRQ*	candidate gene	
X	*GLRA2*	candidate gene	
X	*GPR112*	candidate gene	
X	*GPRASP1*	candidate gene	
X	*HAUS7*	candidate gene	
X	*HDHD1*	candidate gene	
X	*HS6ST2*	candidate gene	
X	*IL3RA*	candidate gene	
X	*ITIH6*	candidate gene	
X	*KCND1*	candidate gene	
X	*KIF4A*	candidate gene	
X	*KLHL15*	candidate gene	
X	*KLHL34*	candidate gene	
X	*KLHL4*	candidate gene	
X	*LAS1L*	candidate gene	
X	*MAGEA11*	candidate gene	
X	*MAGEB1*	candidate gene	
X	*MAGEB10*	candidate gene	
X	*MAGEB2*	candidate gene	
X	*MAGEC1*	candidate gene	
X	*MAGEC3*	candidate gene	
X	*MAGED1*	candidate gene	
X	*MAGEE2*	candidate gene	
X	*MAGIX*	candidate gene	
X	*MAP3K15*	candidate gene	
X	*MAP7D3*	candidate gene	
X	*MBNL3*	candidate gene	
X	*MORC4*	candidate gene	
X	*MSL3*	candidate gene	
X	*MTMR1*	candidate gene	
X	*MTMR8*	candidate gene	
X	*MXRA5*	candidate gene	
X	*NA*	candidate gene	
X	*NKAP*	candidate gene	
X	*NRK*	candidate gene	
X	*NXF4*	candidate gene	
X	*OGT*	candidate gene	
X	*P2RY4*	candidate gene	
X	*P2RY8*	candidate gene	

Chromosome	Gene ID	Known or a Candidate Gene	Origin
X	*PABPC5*	candidate gene	
X	*PASD1*	candidate gene	
X	*PHKA1*	candidate gene	
X	*PIN4*	candidate gene	
X	*PJA1*	candidate gene	
X	*PLCXD1*	candidate gene	
X	*PLXNB3*	candidate gene	
X	*POLA1*	candidate gene	
X	*PRDX4*	candidate gene	
X	*PRICKLE3*	candidate gene	
X	*PRRG1*	candidate gene	
X	*PRRG3*	candidate gene	
X	*PSMD10*	candidate gene	
X	*RENBP*	candidate gene	
X	*RGAG1*	candidate gene	
X	*RGN*	candidate gene	
X	*RLIM*	candidate gene	
X	*RPGR*	candidate gene	
X	*SHROOM2*	candidate gene	
X	*SLC25A53*	candidate gene	
X	*SLC25A6*	candidate gene	
X	*SPRY3*	candidate gene	
X	*STARD8*	candidate gene	
X	*SYTL4*	candidate gene	
X	*TBC1D8B*	candidate gene	
X	*TCEAL3*	candidate gene	
X	*TKTL1*	candidate gene	
X	*TLR8*	candidate gene	
X	*TREX2*	candidate gene	
X	*TSC22D3*	candidate gene	
X	*USP27X*	candidate gene	
X	*UTP14A*	candidate gene	
X	*VAMP7*	candidate gene	
X	*WWC3*	candidate gene	
X	*XIAP*	candidate gene	
X	*XKRX*	candidate gene	
X	*ZFX*	candidate gene	

References for the DDG2P list- [The Deciphering Developmental Disorders Study, 2014]; Gilissen et al. 2014- [Gilissen et al., 2014].

Supp. Table S2. All rare, coding SNPs and indels identified in this study, including non-pathogenic variants

These data are based on the GRCh37/hg19 version of the reference genome. The variants pass quality control and variant frequency metrics as explained in the Materials and Methods and Results sections. Functional annotations were added with the Ensembl Variant Effect Predictor 2.8 against Ensembl 70, and, where the variant affects multiple transcripts, the most severe consequence is reported here, which is not necessarily the canonical gene transcript corresponding to the GenBank mRNA NCBI Reference Sequence. Most of these variants are of unknown significance/likely not to be pathogenic. The likely pathogenic variants, inferred based on the criteria described in the Materials and Methods and the Results sections of the article are in Supp. Tables S3 and S4, as well as in this table. GERP- Genomic Evolutionary Rate Profiling conservation score [Cooper et al., 2005]; Polyphen- tool for annotating coding nonsynonymous SNPs [Adzhubei et al., 2010]; SIFT- tool for predicting whether an amino acid substitution affects protein function [Kumar et al., 2009]; Condel- CONsensus DELeteriousness score of non-synonymous single nucleotide variants [Clifford et al., 2004]; Sample ID- Unique identifier of the studied individuals.

Supp. Table S2 is available as a separate Excel file under the Supporting Information for this article.

Supp. Table S3. Likely causative LoF variants

Chromosome	Genomic DNA Position (hg19 coordinates)	Reference Allele	Alternate allele	Genotype	Effect Variant	NCBI rsID	Gene	Ensembl Transcript	HGVSc Coding DNA Variant Description (Generated with Mutalyzer 2.0.8)	HGVSp Protein Variant Description (Generated with Mutalyzer 2.0.8)	Position in Coding Sequence	Amino Acid Position	HGMD	Pathogenicity Assesment	Frequency in ExAC data [Number alleles with the alternate variant/how many individuals in total]	Frequency NHLBI Exome Variant Server	Sample ID	Gender
1	155408842	G	A	G/A	stop_gained		*ASH1L*	ENST00000392403	NM_018489.2(ASH1L_v001):c.5104C>T	NM_018489.2(ASH1L_i001):p.(Arg1702*)	5104	1702		candidate, in Table 4 main text	0	0	UK10K_FINDWGA5411302	male
X	54099713	G	A	A/A	stop_gained		*FAM120C*	ENST00000328235	XM_005262021.1(FAM120C_v001):c.2632C>T	XM_005262021.1(FAM120C_i001):p.(Gln878*)	2632	878		candidate, in Table 4 main text	0	0	UK10K_FINDWGA5410866	male
6	170110331	C	T	C/T	splice_donor_variant		*PHF10*	ENST00000339209	NM_018288.3:c.1113+1G>A		0	0		candidate, in Table 4 main text	0	0	UK10K_FINDWGA5411045	male
6	170119016	T	G	T/G	splice_acceptor_variant		*PHF10*	ENST00000339209	NM_018288.3:c.195-2A>C		0	0		candidate, in Table 4 main text	0	0	UK10K_FINDWGA5411632	male
6	79727244	CA	C	CA/C	frameshift_variant		*PHIP*	ENST00000275034	NM_017934.5(PHIP_v001):c.1050del	NM_017934.5(PHIP_i001):p.(Phe350Leufs*32)	1050-1051	350-351		candidate, in Table 4 main text	0	0	UK10K_FINDWGA5411669	male
19	13226256	G	GA	G/GA	frameshift_variant		*TRMT1*	ENST00000221504	NM_001142554.1(TRMT1_v001):c.477_478insA	NM_001142554.1(TRMT1_i001):p.(Leu160Thrfs*42)	477-478	159-160		candidate, in Table 4 main text	0	0	UK10K_FINDWGA5410941	male
19	13226262	C	CA	C/CA	frameshift_variant		*TRMT1*	ENST00000221504	NM_001142554.1(TRMT1_v001):c.471_472insA	NM_001142554.1(TRMT1_i001):p.(Glu158Argfs*44)	471-472	157-158		candidate, in Table 4 main text	0	0	UK10K_FINDWGA5410941	male
10	28884798	CA	C	CA/C	frameshift_variant		*WAC*	ENST00000354911	NM_016628.4(WAC_v001):c.748del	NM_016628.4(WAC_i001):p.(Ile250Serfs*81)	748	250		candidate, in Table 4 main text	0	0	UK10K_FINDWGA5410715	male
X	54334354	C	T	T/T	splice_donor_variant		*WNK3*	ENST00000354646	NM_020922.3:c.1089+1G>A		0	0		candidate, in Table 4 main text	0	0	UK10K_FINDWGA5410823	male
1	35453525	A	T	A/T	stop_gained		*ZMYM6*	ENST00000357182	NM_007167.3(ZMYM6_v001):c.3158T>A	NM_007167.3(ZMYM6_i001):p.(Leu1053*)	3158	1053		candidate, in Table 4 main text	0	0	UK10K_FINDWGA5411034	male
1	35453525	A	T	A/T	stop_gained		*ZMYM6*	ENST00000357182	NM_007167.3(ZMYM6_v001):c.3158T>A	NM_007167.3(ZMYM6_i001):p.(Leu1053*)	3158	1053		candidate, in Table 4 main text	0	0	UK10K_FINDWGA5411054	male
X	108902601	G	A	A/A	stop_gained		*ACSL4*	ENST00000340800	NM_022977.2(ACSL4_v001):c.1960C>T	NM_022977.2(ACSL4_i001):p.(Arg654*)	1960	654		likely pathogenic, in Table 1 main text	0	0	UK10K_FINDWGA5411400	male
X	148049184	C	T	T/T	stop_gained		*AFF2*	ENST00000286437	NM_001170628.1(AFF2_v001):c.2152C>T	NM_001170628.1(AFF2_i001):p.(Gln718*)	2152	718		likely pathogenic, in Table 1 main text	0	0	UK10K_FINDWGA5411410	male
4	178359903	C	T	T/T	stop_gained		*AGA*	ENST00000264595	NM_000027.3(AGA_v001):c.503G>A	NM_000027.3(AGA_i001):p.(Trp168*)	503	168	Aspartylglucosaminuria	likely pathogenic, in Table 1 main text	1/116078	0	UK10K_FINDWGA5411606	male
6	157454310	C	G	C/G	stop_gained		*ARID1B*	ENST00000350026	NM_017519.2(ARID1B_v001):c.2481C>G	NM_017519.2(ARID1B_i001):p.(Tyr827*)	2481	827		likely pathogenic, in Table 1 main text	0	0	UK10K_FINDWGA5410663	male
6	157502190	C	T	C/T	stop_gained		*ARID1B*	ENST00000350026	NM_017519.2(ARID1B_v001):c.3184C>T	NM_017519.2(ARID1B_i001):p.(Arg1062*)	3184	1062	Coffin-Siris syndrome	likely pathogenic, in Table 1 main text	0	0	UK10K_FINDWGA5411529	male
6	157495156	GC	G	GC/G	frameshift_variant		*ARID1B*	ENST00000346085	NM_020732.3(ARID1B_v001):c.3041del	NM_020732.3(ARID1B_i001):p.(Ala1014Glufs*3)	3040-3041	1014		likely pathogenic, in Table 1 main text	0	0	UK10K_FINDWGA5411501	male
6	157527816	T	TC	T/TC	frameshift_variant		*ARID1B*	ENST00000350026	NM_017519.2(ARID1B_v001):c.5508dup	NM_017519.2(ARID1B_i001):p.(Ser1838Lysfs*5)	5508	1838		likely pathogenic, in Table 1 main text	0	0	UK10K_FINDWGA5411495	male
20	31021130	C	T	C/T	stop_gained		*ASXL1*	ENST00000375687	NM_015338.5(ASXL1_v001):c.1129C>T	NM_015338.5(ASXL1_i001):p.(Gln377*)	1129	377		likely pathogenic, in Table 1 main text	0	0	UK10K_FINDWGA5411037	female
X	76953099	TG	T	T/T	frameshift_variant		*ATRX*	ENST00000395603	NM_138270.2(ATRX_v001):c.213del	NM_138270.2(ATRX_i001):p.(Lys72Serfs*15)	96	32		likely pathogenic, in Table 1 main text	0	0	UK10K_FINDWGA5411056	male
X	76972632	G	A	A/A	stop_gained	rs122445108	*ATRX*	ENST00000373344	NM_000489.4(ATRX_v001):c.109C>T	NM_000489.4(ATRX_i001):p.(Arg37*)	109	37	ATRX syndrome	likely pathogenic, in Table 1 main text	0	0	UK10K_FINDWGA5411171	male
X	76972632	G	A	A/A	stop_gained	rs122445108	*ATRX*	ENST00000373344	NM_000489.4(ATRX_v001):c.109C>T	NM_000489.4(ATRX_i001):p.(Arg37*)	109	37	ATRX syndrome	likely pathogenic, in Table 1 main text	0	0	UK10K_FINDWGA5410920	male
X	76777868	T	C	C/C	splice_acceptor_variant		*ATRX*	ENST00000373344	NM_000489.4:c.6850-2A>G		0	0		likely pathogenic, in Table 1 main text	0	0	UK10K_FINDWGA5411357	male
X	76954083	C	CAT	CAT/CAT	frameshift_variant		*ATRX*	ENST00000373344	NM_000489.4(ATRX_v001):c.166_167dup	NM_000489.4(ATRX_i001):p.(Met56Ilefs*2)	166-167	56		likely pathogenic, in Table 1 main text	0	0	UK10K_FINDWGA5411068	male
X	76972632	G	A	A/A	stop_gained	rs122445108	*ATRX*	ENST00000373344	NM_000489.4(ATRX_v001):c.109C>T	NM_000489.4(ATRX_i001):p.(Arg37*)	109	37	ATRX syndrome	likely pathogenic, in Table 1 main text	0	0	UK10K_FINDWGA5411170	male
X	79999648	A	T	T/T	stop_gained		*BRWD3*	ENST00000373275	NM_153252.4(BRWD3_v001):c.696T>A	NM_153252.4(BRWD3_i001):p.(Tyr232*)	696	232		likely pathogenic, in Table 1 main text	0	0	UK10K_FINDWGA5410888	male
X	79945282	AC	A	A/A	frameshift_variant		*BRWD3*	ENST00000373275	NM_153252.4(BRWD3_v001):c.3791del	NM_153252.4(BRWD3_i001):p.(Ser1264Ilefs*13)	3791	1264		likely pathogenic, in Table 1 main text	0	0	UK10K_FINDWGA5410935	male
8	61714153	G	T	G/T	splice_donor_variant		*CHD7*	ENST00000423902	NM_017780.3:c.2442+1G>T		0	0		likely pathogenic, in Table 1 main text	0	0	UK10K_FINDWGA5411365	male
8	61763879	G	T	G/T	splice_donor_variant		*CHD7*	ENST00000423902	NM_017780.3:c.5665+1G>T		0	0		likely pathogenic, in Table 1 main text	0	0	UK10K_FINDWGA5411541	male
3	41267250	GC	G	GC/G	frameshift_variant		*CTNNB1*	ENST00000349496	NM_001904.3(CTNNB1_v001):c.835del	NM_001904.3(CTNNB1_i001):p.(Leu279Cysfs*26)	835	279		likely pathogenic, in Table 1 main text	0	0	UK10K_FINDWGA5410768	male
3	41275708	C	T	C/T	stop_gained		*CTNNB1*	ENST00000349496	NM_001904.3(CTNNB1_v001):c.1603C>T	NM_001904.3(CTNNB1_i001):p.(Arg535*)	1603	535		likely pathogenic, in Table 1 main text	0	0	UK10K_FINDWGA5411300	male
X	119680444	T	TA	TA/TA	frameshift_variant		*CUL4B*	ENST00000371322	NM_001079872.1(CUL4B_v001):c.803dup	NM_001079872.1(CUL4B_i001):p.(Leu268Phefs*5)	803	268		likely pathogenic, in Table 1 main text	0	0	UK10K_FINDWGA5410685	male
X	119668443	T	C	C/C	splice_acceptor_variant		*CUL4B*	ENST00000371322	NM_001079872.1:c.2161-2A>G		0	0		likely pathogenic, in Table 1 main text	0	0	UK10K_FINDWGA5411353	male
X	119675504	G	A	A/A	stop_gained		*CUL4B*	ENST00000371322	NM_001079872.1(CUL4B_v001):c.1396C>T	NM_001079872.1(CUL4B_i001):p.(Arg466*)	1396	466		likely pathogenic, in Table 1 main text	0	0	UK10K_FINDWGA5410803	male
X	119694060	A	T	T/T	stop_gained		*CUL4B*	ENST00000371322	NM_001079872.1(CUL4B_v001):c.434T>A	NM_001079872.1(CUL4B_i001):p.(Leu145*)	434	145		likely pathogenic, in Table 1 main text	0	0	UK10K_FINDWGA5411451	male
X	119675472	C	T	T/T	stop_gained		*CUL4B*	ENST00000371322	NM_001079872.1(CUL4B_v001):c.1428G>A	NM_001079872.1(CUL4B_i001):p.(Trp476*)	1428	476		likely pathogenic, in Table 1 main text	0	0	UK10K_FINDWGA5411038	male
9	140707923	GTC	G	GTC/G	frameshift_variant		*EHMT1*	ENST00000460843	NM_024757.4(EHMT1_v001):c.3126_3127del	NM_024757.4(EHMT1_i001):p.(Gln1043Glufs*133)	3126-3127	1043		likely pathogenic, in Table 1 main text	0	0	UK10K_FINDWGA5411373	male
3	71050212	T	C	T/C	splice_acceptor_variant		*FOXP1*	ENST00000318789	NM_001244810.1:c.975-2T>C		0	0		likely pathogenic, in Table 1 main text	0	0	UK10K_FINDWGA5411548	female
X	132826445	AC	A	A/A	frameshift_variant		*GPC3*	ENST00000370818	NM_004484.3(GPC3_v001):c.1243del	NM_004484.3(GPC3_i001):p.(Val415Trpfs*27)	1243	415		likely pathogenic, in Table 1 main text	0	0	UK10K_FINDWGA5411358	male
12	13906612	G	A	G/A	stop_gained		*GRIN2B*	ENST00000279593	NM_000834.3(GRIN2B_v001):c.649C>T	NM_000834.3(GRIN2B_i001):p.(Gln217*)	649	217		likely pathogenic, in Table 1 main text	0	0	UK10K_FINDWGA5411446	male
12	13828715	CA	C	CA/C	frameshift_variant		*GRIN2B*	ENST00000279593	NM_000834.3(GRIN2B_v001):c.1088del	NM_000834.3(GRIN2B_i001):p.(Val363Glyfs*2)	1088	363		likely pathogenic, in Table 1 main text	0	0	UK10K_FINDWGA5411556	female
15	72647903	G	A	G/A	stop_gained	rs121907962	*HEXA*	ENST00000268097	NM_000520.4(HEXA_v001):c.409C>T	NM_000520.4(HEXA_i001):p.(Arg137*)	409	137	Tay-Sachs disease	likely pathogenic, in Table 1 main text	2/122858	0	UK10K_FINDWGA5411084	male
8	43002207	G	A	A/A	splice_donor_variant		*HGSNAT*	ENST00000379644	NM_152419.2: c.234+1G>A		0	0	Mucopolysaccharidosis IIIC	likely pathogenic, in Table 1 main text	3/116352 (none homozygous)	0	UK10K_FINDWGA5411534	female
X	29935691	C	T	T/T	stop_gained		*IL1RAPL1*	ENST00000378993	NM_014271.3(IL1RAPL1_v001):c.889C>T	NM_014271.3(IL1RAPL1_i001):p.(Arg297*)	889	297		likely pathogenic, in Table 1 main text	0	0	UK10K_FINDWGA5410964	male
X	29417326	C	CT	CT/CT	frameshift_variant		*IL1RAPL1*	ENST00000378993	NM_014271.3(IL1RAPL1_v001):c.606dup	NM_014271.3(IL1RAPL1_i001):p.(Ile203Tyrfs*8)	606	203		likely pathogenic, in Table 1 main text	0	0	UK10K_FINDWGA5411107	male
X	29301120	C	T	T/T	stop_gained		*IL1RAPL1*	ENST00000378993	NM_014271.3(IL1RAPL1_v001):c.148C>T	NM_014271.3(IL1RAPL1_i001):p.(Arg50*)	148	50		likely pathogenic, in Table 1 main text	0	0	UK10K_FINDWGA5410971	male
9	745218	C	T	C/T	stop_gained		*KANK1*	ENST00000382297	NM_015158.3(KANK1_v001):c.4042C>T	NM_015158.3(KANK1_i001):p.(Arg1348*)	4042	1348		likely pathogenic, in Table 1 main text	2/122962	0	UK10K_FINDWGA5410746	male
9	713465	G	A	G/A	splice_donor_variant		*KANK1*	ENST00000382303	NM_001256876.1:c.2698+1G>A		0	0		likely pathogenic, in Table 1 main text	0	0	UK10K_FINDWGA5411454	male
10	76729776	A	G	A/G	splice_acceptor_variant		*KAT6B*	ENST00000287239	NM_001256468.1:c.847-2A>G		0	0		likely pathogenic, in Table 1 main text	0	0	UK10K_FINDWGA5410894	male
10	76781026	C	T	C/T	stop_gained		*KAT6B*	ENST00000287239	NM_012330.3(KAT6B_v001):c.3004C>T	NM_012330.3(KAT6B_i001):p.(Arg1002*)	3004	1002		likely pathogenic, in Table 1 main text	0	0	UK10K_FINDWGA5411456	male
X	53240810	C	A	A/A	stop_gained		*KDM5C*	ENST00000452825	NM_001146702.1(KDM5C_v001):c.1069G>T	NM_001146702.1(KDM5C_i001):p.(Glu357*)	1069	357		likely pathogenic, in Table 1 main text	0	0	UK10K_FINDWGA5410821	male
X	43590591	C	CT	CT/CT	frameshift_variant		*MAOA*	ENST00000338702	NM_000240.3(MAOA_v001):c.749_750insT	NM_000240.3(MAOA_i001):p.(Ser251Lysfs*2)	749-750	250		likely pathogenic, in Table 1 main text	0	0	UK10K_FINDWGA5411323	male
5	88024435	CAG	C	CAG/C	frameshift_variant		*MEF2C*	ENST00000340208	NM_001193347.1(MEF2C_v001):c.1003_1004del	NM_001193347.1(MEF2C_i001):p.(Leu335Glufs*2)	1003-1004	335		likely pathogenic, in Table 1 main text	0	0	UK10K_FINDWGA5410689	male
12	49416554	G	GA	G/GA	frameshift_variant		*MLL2*	ENST00000301067	NM_003482.3(KMT2D_v001):c.16157dup	NM_003482.3(KMT2D_i001):p.(Gln5387Serfs*72)	16156-16157	5386	Kabuki syndrome	likely pathogenic, in Table 1 main text	0	0	UK10K_FINDWGA5410982	male
X	6069052	G	C	C/C	stop_gained		*NLGN4X*	ENST00000275857	NM_020742.3(NLGN4X_v001):c.456C>G	NM_020742.3(NLGN4X_i001):p.(Tyr152*)	456	152		likely pathogenic, in Table 1 main text	0	0	UK10K_FINDWGA5411278	male
5	176639197	G	A	G/A	splice_donor_variant		*NSD1*	ENST00000439151	NM_022455.4:c.3796+1G>A		0	0		likely pathogenic, in Table 1 main text	0	0	UK10K_FINDWGA5411100	male
X	13785284	GA	G	GA/G	frameshift_variant		*OFD1*	ENST00000340096	NM_003611.2(OFD1_v001):c.2644del	NM_003611.2(OFD1_i001):p.(Ile882Tyrfs*6)	2639	880		likely pathogenic, in Table 1 main text	0	0	UK10K_FINDWGA5411008	female
X	67414306	C	T	T/T	splice_donor_variant		*OPHN1*	ENST00000355520	NM_002547.2:c.1138+1C>T		0	0		likely pathogenic, in Table 1 main text	0	0	UK10K_FINDWGA5411200	male
X	67331733	G	A	A/A	stop_gained		*OPHN1*	ENST00000355520	NM_002547.2(OPHN1_v001):c.1489C>T	NM_002547.2(OPHN1_i001):p.(Arg497*)	1489	497		likely pathogenic, in Table 1 main text	0	0	UK10K_FINDWGA5411352	male
12	103245464	C	T	T/T	splice_donor_variant	rs62514956	*PAH*	ENST00000307000	NM_000277.1: c.912+1G>A		0	0	Phenylketonuria	likely pathogenic, in Table 1 main text	4/122488 (none homozygous)	0.000154	UK10K_FINDWGA5411524	female
11	31815627	G	A	G/A	stop_gained	rs121907917	*PAX6*	ENST00000379123	NM_001258464.1: c.718C>T	NM_001258464.1(PAX6_i001):p.(Arg240*)	718	240	Aniridia	likely pathogenic, in Table 1 main text	0	0	UK10K_FINDWGA5410895	male
X	48759320	G	A	A/A	splice_donor_variant		*PQBP1*	ENST00000218224	NM_005710.2:c.292+1G>A		0	0		likely pathogenic, in Table 1 main text	0	0	UK10K_FINDWGA5411452	male
X	48759667	CAGAG	C	C/C	frameshift_variant		*PQBP1*	ENST00000218224	NM_005710.2(PQBP1_v001):c.459_462del	NM_005710.2(PQBP1_i001):p.(Arg153Serfs*41)	451-454	151-152		likely pathogenic, in Table 1 main text	0	0	UK10K_FINDWGA5410844	male

Supp. Table S3. Likely causative LoF variants cont.

Chromosome	Genomic DNA Position (hg19 coordinates)	Reference Allele	Alternate allele	Genotype	Effect variant	NCBI rsID	Gene	Ensembl Transcript	HGVSc Coding DNA Variant Description (Generated with Mutalyzer 2.0.8)	HGVSp protein variant description (generated with Mutalyzer 2.0.8)	Position in coding sequence	Amino acid position	HGMD	Pathogenicity assesment	Frequency in ExAC data [Number alleles with the alternate variant/how many individuals in total]	Frequency NHLBI Exome Variant Server	Sample ID	Gender
X	23411323	CTA	C	C/C	frameshift_variant		*PTCHD1*	ENST00000379361	NM_173495.2(PTCHD1_v001):c.1691_1692del	NM_173495.2(PTCHD1_i001):p.(Ile564Argfs*6)	1689-1690	563-564		likely pathogenic, in Table 1 main text	0	0	UK10K_FINDWGA5410926	male
10	89717672	C	T	C/T	stop_gained	rs121909219	*PTEN*	ENST00000371953	NM_000314.6(PTEN_v001):c.697C>T	NM_000314.6(PTEN_i001):p.(Arg233*)	697	233	Cowden disease	likely pathogenic, in Table 1 main text	0	0	UK10K_FINDWGA5411535	male
2	166198965	C	T	C/T	stop_gained		*SCN2A*	ENST00000283256	NM_021007.2(SCN2A_v001):c.2548C>T	NM_021007.2(SCN2A_i001):p.(Arg850*)	2548	850		likely pathogenic, in Table 1 main text	0	0	UK10K_FINDWGA5410774	male
2	166179816	A	T	A/T	stop_gained		*SCN2A*	ENST00000283256	NM_021007.2(SCN2A_v001):c.1822A>T	NM_021007.2(SCN2A_i001):p.(Arg608*)	1822	608		likely pathogenic, in Table 1 main text	0	0	UK10K_FINDWGA5410691	male
12	52156446	C	T	C/T	stop_gained		*SCN8A*	ENST00000354534	NM_001177984.2(SCN8A_v001):c.2530C>T	NM_001177984.2(SCN8A_i001):p.(Arg844*)	2530	844		likely pathogenic, in Table 1 main text	0	0	UK10K_FINDWGA5411661	male
18	42531178	C	T	C/T	stop_gained		*SETBP1*	ENST00000282030	NM_015559.2(SETBP1_v001):c.1873C>T	NM_015559.2(SETBP1_i001):p.(Arg625*)	1873	625		likely pathogenic, in Table 1 main text	0	0	UK10K_FINDWGA5411268	male
3	9486739	A	T	A/T	stop_gained		*SETD5*	ENST00000402198	NM_001080517.1(SETD5_v001):c.1195A>T	NM_001080517.1(SETD5_i001):p.(Lys399*)	1195	399		likely pathogenic, in Table 1 main text	0	0	UK10K_FINDWGA5411029	male
3	9517301	CT	C	CT/C	frameshift_variant		*SETD5*	ENST00000402198	NM_001080517.1(SETD5_v001):c.3856del	NM_001080517.1(SETD5_i001):p.(Ser1286Leufs*84)	3856	1286		likely pathogenic, in Table 1 main text	0	0	UK10K_FINDWGA5410795	male
3	9486877	C	T	C/T	stop_gained		*SETD5*	ENST00000402198	NM_001080517.1(SETD5_v001):c.1333C>T	NM_001080517.1(SETD5_i001):p.(Arg445*)	1333	445		likely pathogenic, in Table 1 main text	0	0	UK10K_FINDWGA5411659	male
3	9517216	A	AG	A/AG	frameshift_variant		*SETD5*	ENST00000402198	NM_001080517.1(SETD5_v001):c.3771dup	NM_001080517.1(SETD5_i001):p.(Ser1258Glufs*65)	3771	1258		likely pathogenic, in Table 1 main text	0	0	UK10K_FINDWGA5411248	male
3	9512419	C	T	C/T	stop_gained		*SETD5*	ENST00000402198	NM_001080517.1(SETD5_v001):c.3001C>T	NM_001080517.1(SETD5_i001):p.(Arg1001*)	3001	1001		likely pathogenic, in Table 1 main text	0	0	UK10K_FINDWGA5411283	male
3	9489453	C	G	C/G	stop_gained		*SETD5*	ENST00000402198	NM_001080517.1(SETD5_v001):c.1866C>G	NM_001080517.1(SETD5_i001):p.(Tyr622*)	1866	622		likely pathogenic, in Table 1 main text	0	0	UK10K_FINDWGA5410802	male
3	9490142	TCA	T	TCA/T	frameshift_variant		*SETD5*	ENST00000402198	NM_001080517.1(SETD5_v001):c.2177_2178del	NM_001080517.1(SETD5_i001):p.(Thr726Asnfs*39)	2177-2178	726		likely pathogenic, in Table 1 main text	0	0	UK10K_FINDWGA5410830	male
11	70332413	G	A	G/A	stop_gained		*SHANK2*	ENST00000449833	NM_133266.3(SHANK2_v001):c.2221C>T	NM_133266.3(SHANK2_i001):p.(Gln741*)	2221	741		likely pathogenic, in Table 1 main text	0	0	UK10K_FINDWGA5411435	male
X	135106576	TTG	T	T/T	frameshift_variant		*SLC9A6*	ENST00000370695	NM_001042537.1(SLC9A6_v001):c.1554_1555del	NM_001042537.1(SLC9A6_i001):p.(Phe520Tyrfs*23)	1554-1555	520		likely pathogenic, in Table 1 main text	0	0	UK10K_FINDWGA5411275	male
X	135081128	G	A	A/A	splice_donor_variant		*SLC9A6*	ENST00000370695	NM_001042537.1:c.793+1G>A		0	0		likely pathogenic, in Table 1 main text	0	0	UK10K_FINDWGA5410836	male
18	52946888	C	T	C/T	splice_acceptor_variant		*TCF4*	ENST00000354452	NM_001083962.1:c.550-1G>A		0	0	Pitt-Hopkins syndrome	likely pathogenic, in Table 1 main text	0	0	UK10K_FINDWGA5411380	male
18	53017634	G	A	G/A	stop_gained		*TCF4*	ENST00000354452	NM_001083962.1(TCF4_v001):c.505C>T	NM_001083962.1(TCF4_i001):p.(Gln169*)	505	169		likely pathogenic, in Table 1 main text	0	0	UK10K_FINDWGA5410771	male
X	38525419	T	TA	TA/TA	frameshift_variant		*TSPAN7*	ENST00000378482	NM_004615.3(TSPAN7_v001):c.127dup	NM_004615.3(TSPAN7_i001):p.(Thr43Asnfs*42)	127	43		likely pathogenic, in Table 1 main text	0	0	UK10K_FINDWGA5410923	male
15	25601177	TA	T	TA/T	frameshift_variant		*UBE3A*	ENST00000232165	NM_000462.3(UBE3A_v001):c.1995del	NM_000462.3(UBE3A_i001):p.(Leu665Phefs*12)	1995	665		likely pathogenic, in Table 1 main text	0	0	UK10K_FINDWGA5411225	male
X	118985742	G	GA	GA/GA	frameshift_variant		*UPF3B*	ENST00000276201	NM_080632.2(UPF3B_v001):c.250del	NM_080632.2(UPF3B_i001):p.(Ser84Leufs*47)	250-251	84		likely pathogenic, in Table 1 main text	0	0	UK10K_FINDWGA5410747	male
X	118975080	TTCTG	T	T/T	frameshift_variant		*UPF3B*	ENST00000276201	NM_080632.2(UPF3B_v001):c.762_765del	NM_080632.2(UPF3B_i001):p.(Asp254Glufs*8)	762-765	254-255		likely pathogenic, in Table 1 main text	0	0	UK10K_FINDWGA5411299	male
X	41089847	CA	C	C/C	frameshift_variant		*USP9X*	ENST00000324545	NM_001039590.2(USP9X_v001):c.7574del	NM_001039590.2(USP9X_i001):p.(Gln2525Argfs*18)	7574	2525		likely pathogenic, in Table 1 main text	0	0	UK10K_FINDWGA5411140	male
X	128944967	G	A	A/A	stop_gained		*ZDHHC9*	ENST00000357166	NM_016032.3(ZDHHC9_v001):c.892C>T	NM_016032.3(ZDHHC9_i001):p.(Arg298*)	892	298	ID	likely pathogenic, in Table 1 main text	0	0	UK10K_FINDWGA5410775	male
X	128945384	G	GA	GA/GA	frameshift_variant		*ZDHHC9*	ENST00000357166	NM_016032.3(ZDHHC9_v001):c.878_879insA	NM_016032.3(ZDHHC9_i001):p.(Ser294Glnfs*26)	878-879	293		likely pathogenic, in Table 1 main text	0	0	UK10K_FINDWGA5410704	male

The genomic position of the variants is provided according to the GRCh37/hg19 version of the reference genome; NCBI rsID- reference number if the variant has been annotated in the NCBI dbSNP database; HGVSc Coding DNA Variant Description- the nucleotide numbering uses +1 as the A of the ATG translation initiation codon in the reference sequence, with the initiation codon as codon 1, the description has been generated with Mutalyzer 2.0.8; the HGVSp Protein Variant Description has been generated with Mutalyzer 2.0.8 [Wildeman et al., 2008]; HGMD - annotated if the variant has been observed in the HGMD Professional 2014.3 version [Stenson et al., 2014]; Frequency in ExAC data [Number alleles with the alternate variant/how many individuals in total]- frequency data from the Exome Aggregation Consortium ((ExAC), Cambridge, MA; URL: http://exac.broadinstitute.org; accessed November 2014); Frequency NHLBI Exome Variant Server-frequency data from the NHLBI Exome Sequencing Project (Exome Variant Server (EVS), http://evs.gs.washington.edu/EVS/) [Tabor et al., 2014]; Sample ID- Unique identifier of the studied individuals.

Supp. Table S4. Likely causative missense variants

Chromosome	Genomic DNA Position (hg19 coordinates)	Reference Allele	Alternate Allele	Genotype	Effect Variant	NCBI rsID	Gene	Ensembl Transcript	HGVSc Coding DNA Variant Description (Generated with Mutalyzer 2.0.8)	HGVSp Protein Variant Description (Generated with Mutalyzer 2.0.8)	Position in Coding Sequence	Amino Acid Position	Aminoacid Change	HGMD	Pathogenicity Assesment	Frequency in ExAC data [Number alleles with the alternate variant/how many individuals in total]	Frequency NHLBI Exome Variant Server	GERP	PolyPhen	SIFT	Condel	Sample ID	Gender
6	157431632	G	A	G/A	missense_variant	.	*ARID1B*	ENST00000346085	NM_020732.3(ARID1B_v001):c.2308G>A	NM_020732.3(ARID1B_i001):p.(Gly770Arg)	2308	770	G>R	Intellectual disability, plantar fat pads & facial dysmorphism, not exactly the same variant, (CCG/-)	likely pathogenic, in Table 3 main text	0	0	2.17	probably_damaging(0.964)	0	0	UK10K_FINDWGA5410858	male
X	76829787	C	T	T/T	missense_variant	.	*ATRX*	ENST00000373344	NM_000489.4(ATRX_v001):c.6254G>A	NM_000489.4(ATRX_i001):p.(Arg2085His)	6254	2085	R>H	ATRX syndrome	likely pathogenic, in Table 3 main text	0	0	3.84	probably_damaging(0.997)	0	0	UK10K_FINDWGA5411356	male
X	76829787	C	T	T/T	missense_variant	.	*ATRX*	ENST00000373344	NM_000489.4(ATRX_v001):c.6254G>A	NM_000489.4(ATRX_i001):p.(Arg2085His)	6254	2085	R>H	ATRX syndrome	likely pathogenic, in Table 3 main text	0	0	3.84	probably_damaging(0.997)	0	0	UK10K_FINDWGA5410693	male
8	61763644	C	T	C/T	missense_variant	.	*CHD7*	ENST00000423902	NM_017780.3(CHD7_v001):c.5588C>T	NM_017780.3(CHD7_i001):p.(Pro1863Leu)	5588	1863	P>L	CHARGE syndrome	likely pathogenic, in Table 3 main text	2/29858	0	3.72	benign(0.021)	tolerated(0.48)	neutral(0.011)	UK10K_FINDWGA5411437	male
7	147964128	G	C	G/C	missense_variant	.	*CNTNAP2*	ENST00000361727	NM_014141.5(CNTNAP2_v001):c.3385G>C	NM_014141.5(CNTNAP2_i001):p.(Asp1129His)	3385	1129	D>H	Autism	likely pathogenic, in Table 3 main text	2/122966	0	3.48	possibly_damaging(0.883)	deleterious(0)	deleterious(0.789)	UK10K_FINDWGA5411520	male
11	71153365	T	C	T/C	missense_variant	.	*DHCR7*	ENST00000355527	NM_001360.2(DHCR7_v001):c.356A>G	NM_001360.2(DHCR7_i001):p.(His119Arg)	356	119	H>R	Smith-Lemli-Opitz syndrome	likely pathogenic, in Table 3 main text	4/116252	0	3.38	probably_damaging(0.946)	deleterious(0)	deleterious(0.835)	UK10K_FINDWGA5411429	male
11	71155134	C	T	C/T	missense_variant	.	*DHCR7*	ENST00000355527	NM_001360.2(DHCR7_v001):c.226G>A	NM_001360.2(DHCR7_i001):p.(Val76Ile)	226	76	V>I		likely pathogenic, in Table 3 main text	195/116466	0	1.57	benign(0.006)	tolerated(0.4)	neutral(0.016)	UK10K_FINDWGA5411429	male
X	31196049	G	A	A/A	missense_variant	rs104894791	*DMD*	ENST00000357033	NM_000109.3(DMD_v001):c.10238C>T	NM_000109.3(DMD_i001):p.(Ala3413Val)	10238	3413	A>V	Muscular dystrophy, Becker	likely pathogenic, in Table 3 main text	0	0	2.86	benign(0.044)	tolerated(0.06)	neutral(0.325)	UK10K_FINDWGA5410830	male
X	122616707	G	A	A/A	missense_variant	rs137852350	*GRIA3*	ENST00000264357	NM_000828.4(GRIA3_v001):c.2497G>A	NM_000828.4(GRIA3_i001):p.(Gly833Arg)	2497	833	G>R	X-linked ID	likely pathogenic, in Table 3 main text	0	0	4.65	probably_damaging(0.999)	deleterious(0)	deleterious(0.935)	UK10K_FINDWGA5411367	male
16	9858474	T	C	T/C	missense_variant	.	*GRIN2A*	ENST00000330684	NM_001134407.2(GRIN2A_v001):c.2927A>G	NM_001134407.2(GRIN2A_i001):p.(Asn976Ser)	2927	976	N>S	Epileptic encephalopathy	likely pathogenic, in Table 3 main text	0	0	1.96	probably_damaging(0.996)	tolerated(0.06)	deleterious(0.784)	UK10K_FINDWGA5411663	male
11	534289	C	T	C/T	missense_variant	rs104894229	*HRAS*	ENST00000451590	NM_001130442.1(HRAS_v001):c.34G>A	NM_001130442.1(HRAS_i001):p.(Gly12Ser)	34	12	G>S	Costello syndrome	likely pathogenic, in Table 3 main text	0	0	3.15	possibly_damaging(0.525)	deleterious(0.02)		UK10K_FINDWGA5411109	female
8	133192493	G	T	G/T	missense_variant	.	*KCNQ3*	ENST00000388996	NM_004519.3(KCNQ3_v001):c.688C>A	NM_004519.3(KCNQ3_i001):p.(Arg230Ser)	688	230	R>S	non-syndromic ID	likely pathogenic, in Table 3 main text	0	0	5.79	probably_damaging(0.965)	deleterious(0)	deleterious(0.851)	UK10K_FINDWGA5410783	female
12	25398279	C	T	C/T	missense_variant	rs104894365	*KRAS*	ENST00000256078	NM_033360.3(KRAS_v001):c.40G>A	NM_033360.3(KRAS_i001):p.(Val14Ile)	40	14	V>I	Noonan syndrome	likely pathogenic, in Table 3 main text	1/103262	0	4.58	probably_damaging(0.977)	deleterious(0)	deleterious(0.863)	UK10K_FINDWGA5411395	male
12	25398279	C	T	C/T	missense_variant	rs104894365	*KRAS*	ENST00000256078	NM_033360.3(KRAS_v001):c.40G>A	NM_033360.3(KRAS_i001):p.(Val14Ile)	40	14	V>I	Noonan syndrome	likely pathogenic, in Table 3 main text	1/103262	0	4.58	probably_damaging(0.977)	deleterious(0)	deleterious(0.863)	UK10K_FINDWGA5411607	male
15	66729181	A	G	A/G	missense_variant	rs121908595	*MAP2K1*	ENST00000307102	NM_002755.3(MAP2K1_v001):c.389A>G	NM_002755.3(MAP2K1_i001):p.(Tyr130Cys)	389	130	Y>C	Cardio-facio-cutaneous syndrome	likely pathogenic, in Table 3 main text	0	0	4.02	probably_damaging(0.998)	deleterious(0)	deleterious(0.919)	UK10K_FINDWGA5411227	male
X	153296780	G	A	A/A	missense_variant	rs61748420	*MECP2*	ENST00000303391	NM_004992.3(MECP2_v001):c.499C>T	NM_004992.3(MECP2_i001):p.(Arg167Trp)	499	167	R>W	X-linked ID	likely pathogenic, in Table 3 main text	0	0	2.8	probably_damaging(0.994)	deleterious(0)	deleterious(0.897)	UK10K_FINDWGA5411184	male
X	153296860	G	A	A/A	missense_variant	rs28934908	*MECP2*	ENST00000303391	NM_004992.3(MECP2_v001):c.419C>T	NM_004992.3(MECP2_i001):p.(Ala140Val)	419	140	A>V	Rett syndrome	likely pathogenic, in Table 3 main text	0	0	3.7	probably_damaging(0.97)	deleterious(0.04)	deleterious(0.761)	UK10K_FINDWGA5410713	male
X	153296811	G	C	G/C	missense_variant	rs61748408	*MECP2*	ENST00000303391	NM_004992.3(MECP2_v001):c.468C>G	NM_004992.3(MECP2_i001):p.(Asp156Glu)	468	156	D>E	Rett syndrome	likely pathogenic, in Table 3 main text	0	0	3.67	probably_damaging(0.99)	deleterious(0)	deleterious(0.886)	UK10K_FINDWGA5411483	female
X	153296725	C	A	A/A	missense_variant	.	*MECP2*	ENST00000303391	NM_004992.3(MECP2_v001):c.554G>T	NM_004992.3(MECP2_i001):p.(Gly185Val)	554	185	G>V	Rett syndrome	likely pathogenic, in Table 3 main text	0	0	3.92	probably_damaging(0.998)	deleterious(0.02)	deleterious(0.857)	UK10K_FINDWGA5411185	male
X	153296662	C	G	G/G	missense_variant	rs63485860	*MECP2*	ENST00000303391	NM_004992.3(MECP2_v001):c.617G>C	NM_004992.3(MECP2_i001):p.(Gly206Ala)	617	206	G>A	Rett syndrome	likely pathogenic, in Table 3 main text	0	0	3.92	probably_damaging(0.989)	deleterious(0.01)	deleterious(0.845)	UK10K_FINDWGA5410887	male
X	70347217	C	T	T/T	missense_variant	rs80338758	*MED12*	ENST00000333646	NM_005120.2(MED12_v001):c.2881C>T	NM_005120.2(MED12_i001):p.(Arg961Trp)	2881	961	R>W	FG syndrome 1	likely pathogenic, in Table 3 main text	0	0	NA	probably_damaging(0.936)	deleterious(0.01)	deleterious(0.787)	UK10K_FINDWGA5411442	male
X	70347217	C	T	T/T	missense_variant	rs80338758	*MED12*	ENST00000333646	NM_005120.2(MED12_v001):c.2881C>T	NM_005120.2(MED12_i001):p.(Arg961Trp)	2881	961	R>W	FG syndrome 1	likely pathogenic, in Table 3 main text	0	0	NA	probably_damaging(0.936)	deleterious(0.01)	deleterious(0.787)	UK10K_FINDWGA5411178	male
5	176638672	T	G	T/G	missense_variant	.	*NSD1*	ENST00000347982	NM_172349.2(NSD1_v001):c.2465T>G	NM_172349.2(NSD1_i001):p.(Leu822Arg)	2465	822	L>R	Autism	likely pathogenic, in Table 3 main text	3/122408	0	1.27	possibly_damaging(0.73)	deleterious(0)	deleterious(0.711)	UK10K_FINDWGA5411386	male
X	110385347	C	T	T/T	missense_variant	rs121434612	*PAK3*	ENST00000262836	NM_001128173.1(PAK3_v001):c.199C>T	NM_001128173.1(PAK3_i001):p.(Arg67Cys)	199	67	R>C	Mental retardation syndrome, X-linked	likely pathogenic, in Table 3 main text	0	0	4.65	probably_damaging(0.964)	deleterious(0)	deleterious(0.851)	UK10K_FINDWGA5410671	male
16	29824904	G	A	G/A	missense_variant	.	*PRRT2*	ENST00000300797	NM_001256443.1(PRRT2_v001):c.529G>A	NM_001256443.1(PRRT2_i001):p.(Glu177Lys)	529	177	E>K	Paroxysmal kinesigenic dyskinesia	likely pathogenic, in Table 3 main text	0	0	2.85	benign(0.275)	deleterious(0.01)	neutral(0.435)	UK10K_FINDWGA5410887	male
10	89653851	T	C	T/C	missense_variant	.	*PTEN*	ENST00000371953	NM_000314.6(PTEN_v001):c.149T>C	NM_000314.6(PTEN_i001):p.(Ile50Thr)	149	50	I>T	yes, DM, Macrocephaly	likely pathogenic, in Table 3 main text	0	0	3.73	probably_damaging(0.979)	deleterious(0)	deleterious(0.867)	UK10K_FINDWGA5411083	male
10	112724120	A	G	A/G	missense_variant	.	*SHOC2*	ENST00000265277	NM_001269039.1(SHOC2_v001):c.4A>G	NM_001269039.1(SHOC2_i001):p.(Ser2Gly)	4	2	S>G	Noonan-like syndrome with loose anagen hair	likely pathogenic, in Table 3 main text	0	0	4.06	probably_damaging(0.925)	deleterious(0)	deleterious(0.818)	UK10K_FINDWGA5411252	male
1	43395364	T	A	T/A	missense_variant	rs148359799	*SLC2A1*	ENST00000426263	NM_006516.2(SLC2A1_v001):c.767A>T	NM_006516.2(SLC2A1_i001):p.(Lys256Met)	767	256	K>M	Glucose transporter 1 deficiency syndrome	likely pathogenic, in Table 3 main text	0	0	2.54	probably_damaging(0.98)	deleterious(0)	deleterious(0.869)	UK10K_FINDWGA5411433	male
1	43395365	T	C	T/C	missense_variant	rs121909738	*SLC2A1*	ENST00000426263	NM_006516.2(SLC2A1_v001):c.766A>G	NM_006516.2(SLC2A1_i001):p.(Lys256Glu)	766	256	K>E	Glucose transporter 1 deficiency syndrome	likely pathogenic, in Table 3 main text	0	0	1.34	possibly_damaging(0.811)	deleterious(0.01)	deleterious(0.711)	UK10K_FINDWGA5411433	male
1	43396718	G	A	G/A	missense_variant	.	*SLC2A1*	ENST00000372500	NM_006516.2(SLC2A1_v001):c.274C>T	NM_006516.2(SLC2A1_i001):p.(Arg92Trp)	274	92	R>W	Paroxysmal dyskinesia, exertion-induced	likely pathogenic, in Table 3 main text	0	0	-2.93	probably_damaging(1)	deleterious(0)	deleterious(0.945)	UK10K_FINDWGA5410904	male
X	21985430	G	A	A/A	missense_variant	rs121434610	*SMS*	ENST00000379404	NM_001258423.1(SMS_v001):c.166G>A	NM_001258423.1(SMS_i001):p.(Gly56Ser)	166	56	G>S	Snyder-Robinson syndrome	likely pathogenic, in Table 3 main text	0	0	4.41	benign(0.132)	tolerated(0.22)	neutral(0.053)	UK10K_FINDWGA5410846	male
9	130444788	C	G	C/G	missense_variant	.	*STXBP1*	ENST00000373299	NM_001032221.3(STXBP1_v001):c.1651C>G	NM_001032221.3(STXBP1_i001):p.(Arg551Gly)	1651	551	R>G	Autism	likely pathogenic, in Table 3 main text	0	0	4.51	probably_damaging(1)	deleterious(0)	deleterious(0.945)	UK10K_FINDWGA5411254	male
12	49578884	C	T	C/T	missense_variant	rs137853050	*TUBA1A*	ENST00000295766	NM_001270399.1(TUBA1A_v001):c.1265G>A	NM_001270399.1(TUBA1A_i001):p.(Arg422His)	1265	422	R>H	Lissencephaly	likely pathogenic, in Table 3 main text	0	0	1.84	benign(0.129)	0	0	UK10K_FINDWGA5410837	male
X	128957700	G	A	A/A	missense_variant	rs137852214	*ZDHHC9*	ENST00000357166	NM_016032.3(ZDHHC9_v001):c.442C>T	NM_016032.3(ZDHHC9_i001):p.(Arg148Trp)	442	148	R>W	X-linked ID	likely pathogenic, in Table 3 main text	0	0	4.64	probably_damaging(0.998)	deleterious(0)	deleterious(0.919)	UK10K_FINDWGA5411590	male
15	72639044	G	A	G/A	missense_variant		*HEXA*	ENST00000268097	NM_000520.4(HEXA_v001):c.1154C>T	NM_000520.4(HEXA_i001):p.(Pro385Leu)	1154	385	P>L		likely pathogenic- individual is compound heterozygote as there is also a LoF variant observed (Table 1 main text)	0	0	NA	possibly_damaging(0.488)	deleterious(0.01)	0	UK10K_FINDWGA5411084	male
8	43025804	C	A	A/A	missense_variant		*HGSNAT*	ENST00000379644	NM_152419.2(HGSNAT_v001):c.710C>A	NM_152419.2(HGSNAT_i001):p.(Pro237Gln)	710	237	P>Q	Mucopolysaccharidosis IIIC	likely pathogenic- individual is compound heterozygote as there is also a LoF variant observed (Table 1 main text)	1/70280 (not homozygous)	0	0.25	benign(0.038)	tolerated(0.42)	neutral(0.016)	UK10K_FINDWGA5411534	female

The genomic position of the variants is provided according to the GRCh37/hg19 version of the reference genome; NCBI rsID- reference number if the variant has been annotated in the NCBI dbSNP database; HGVSc Coding DNA Variant Description- the nucleotide numbering uses +1 as the A of the ATG translation initiation codon in the reference sequence, with the initiation codon as codon 1, the description has been generated with Mutalyzer 2.0.8; the HGVSp Protein Variant Description has been generated with Mutalyzer 2.0.8 [Wildeman et al., 2008]; HGMD- annotated if the variant has been observed in the HGMD Professional 2014.3 version [Stenson et al., 2014]; Frequency in ExAC data [Number alleles with the alternate variant/how many individuals in total]- frequency data from the Exome Aggregation Consortium ((ExAC), Cambridge, MA; URL: http://exac.broadinstitute.org; accessed November 2014); Frequency NHLBI Exome Variant Server-frequency data from the NHLBI Exome Sequencing Project (Exome Variant Server (EVS), http://evs.gs.washington.edu/EVS/) [Tabor et al., 2014]; GERP- Genomic Evolutionary Rate Profiling conservation score [Cooper et al., 2005]; Polyphen- tool for annotating coding nonsynonymous SNPs [Adzhubei et al., 2010]; SIFT- tool for predicting whether an amino acid substitution affects protein function [Kumar et al., 2009]; Condel- CONsensus DELeteriousness score of non-synonymous single nucleotide variants [Clifford et al., 2004]; Sample ID- Unique identifier of the studied individuals.

Supp. Text S1. Consortia Members

Members of the Italian X-linked Mental Retardation Project

G. Strangoni (Assisi), G. D'Avanzo (Avellino), F. Carnevale (Bari), N Resta (Bari), G. Scarano (Benevento), L. Mazzanti (Bologna), R. Borgatti (Bosisio Parini), E. Marchina (Brescia), P. Strisciuglio (Catanzaro), P. Cavalli (Cremona), S. Bigoni (Ferrara), E. Zammarchi (Firenze), F. Faravelli (Genova), M. Di Rocco (Genova Gaslini), M. Lerone (Genova Gaslini), E. D'Alessandro (L'Aquila), A. Selicorni (Monza), C. Pantaleoni (Milano), F. Bedeschi (Milano), MM. Rinaldi (Napoli), R. Tenconi (Padova), A. Verri (Pavia), A. Battaglia (Pisa), R. Guerrini (Firenze), M. Priolo (Reggio Calabria), L. Garavelli (Reggio Emilia), G. Neri (Roma), M. Pergola (Roma), C. Galasso (Roma), L. Zelante (San Giovanni Rotondo), A. Renieri (Siena), G. Ferrero (Torino), L. Memo (Belluno), L. Turolla (Treviso), U. Hladnik (Trieste), C. Romano (Troina).

Members of UK10K Consortium

A full list of consortium members can be found at the UK10K Project website (http://www.uk10k.org).

Members of GOLD Consortium

Clinician	Hospital	City	Country
N Van der Aa	Center for Human Genetics	Leuven	Belgium
A Ahmed	Liverpool Women's NHS Foundation Trust	Liverpool	UK
V K Ajith	St George's University of London	London	UK
H Archer	University Hospital of Wales	Cardiff	UK
R Armstrong	Cambridge University Hospitals, Addenbrooke's hospital	Cambridge	UK
M Balasubramanian	Sheffield Children's Hospital	Sheffield	UK
D Baralle	University Hospital Southampton	Southampton	UK
A Barnicoat	Great Ormond Street Hospital for Children	London	UK
P Beales	Great Ormond Street Hospital for Children	London	UK
C Bennett	The Leeds Teaching Hospitals	Leeds	UK
B Bernhard	North West Thames Regional Genetic Service	Harrow	UK
L Bianciardi	University of Siena	Siena	Italy
M Bitner-Glindzicz	Great Ormond Street Hospital for Children	London	UK
E Blair	Oxford University Hospitals NHS	Oxford	UK

Clinician	Hospital	City	Country
	Trust		
H van Bokhoven	Radboud University Medical Centre	Nijmegen	The Netherlands
B van Bon	Radboud University Medical Centre	Nijmegen	The Netherlands
L Bradley	Belfast City Hospital Trust, Northern Ireland regional Genetics Centre	Belfast	UK
A Brady	North West Thames Regional Genetic Service	Harrow	UK
C Brewer	Peninsula Clinical Genetics Service	Devon & Exeter	UK
H Brunner	Radboud University Medical Centre	Nijmegen	The Netherlands
M Burke	Liverpool Women's NHS Foundation Trust	Liverpool	UK
A Caliebe	University Hospital Schleswig-Holstein Campus Kiel	Kiel	Germany
N Canham	North West Thames Regional Genetic Service	Harrow	UK
B Castle	Peninsula Clinical Genetics Service	Devon & Exeter	UK
K Chandler	Central Manchester University Hospitals	Manchester	UK
A Clarke	University Hospital of Wales	Cardiff	UK
J Clayton-Smith	Central Manchester University Hospitals	Manchester	UK
V Clowes	North West Thames Regional Genetic Service	Harrow	UK
T Cole	Birmingham Women's Hospital	Birmingham	UK
A Collins	University Hospital Southampton	Southampton	UK
J Cook	Sheffield Children's Hospital	Sheffield	UK
C Coughlin	The Children's Hospital of Philadelphia	Philadelphia	USA
A Cowe	University Hospital of Wales	Cardiff	UK
H Cox	Birmingham Women's Hospital	Birmingham	UK
Y Crow	Central Manchester University Hospitals	Manchester	UK
T Dabir	Belfast City Hospital Trust, Northern Ireland regional Genetics Centre	Belfast	UK
S Davies	University Hospital of Wales	Cardiff	UK
D Deshpande	Guy's and St Thomas' Hospital	London	UK
KEM Diderich	Erasmus Medical Centre	Rotterdam	The Netherlands
C Dolling	University Hospitals Bristol, NHS Foundation Trust, St Michael's Hospital	Bristol	UK
D Donnai	Central Manchester University Hospitals	Manchester	UK

Clinician	Hospital	City	Country
D Donnelly	Belfast City Hospital Trust, Northern Ireland regional Genetics Centre	Belfast	UK
D Dooijes	Erasmus Medical Centre	Rotterdam	The Netherlands
J Dupont	Hospital de Santa Maria	Lisbon	Portugal
I Ellis	Liverpool Women's NHS Foundation Trust	Liverpool	UK
H Van Esch	Center for Human Genetics	Leuven	Belgium
M Field	Hunter Genetics	Waratah	Australia
R De Filippis	University of Siena	Siena	Italy
H Firth	Cambridge University Hospitals, Addenbrooke's hospital	Cambridge	UK
R Fisher	The Newcastle upon Tyne Hospitals	Newcastle upon Tyne	UK
D Fitzpatrick	University of Edinburgh Western General Hospital	Edinburgh	UK
N Foulds	University Hospital Southampton	Southampton	UK
B Franco	Università degli Studi di Napoli Federico II	Napoli	Italy
A Fry	University Hospital of Wales	Cardiff	UK
A Fryer	Liverpool Women's NHS Foundation Trust	Liverpool	UK
G Fuchs	University Hospitals of Leicester NHS Trust	Leicester	UK
S Garcia	North West Thames Regional Genetic Service	Harrow	UK
C Gardiner	Nottingham University Hospital NHS Trust	Nottingham	UK
J Gecz	University of Adelaide	Adelaide	Australia
R Gibbons	Oxford University Hospitals NHS Trust	Oxford	UK
J Goodship	The Newcastle upon Tyne Hospitals	Newcastle upon Tyne	UK
A Green	Our Lady's Hospital for Sick Children	Dublin	UK
L Greenhalgh	Liverpool Women's NHS Foundation Trust	Liverpool	UK
G Guanti	University of Bari	Bari	Italy
P Guilbert	Nottingham University Hospital NHS Trust	Nottingham	UK
A Hackett	Hunter Genetics	Waratah	Australia
MV Halest	North West Thames Regional Genetic Service	Harrow	UK
M Haroon	University Hospitals of Leicester NHS Trust	Leicester	UK
J Harvey	University Hospital Southampton	Southampton	UK
A Henderson	The Newcastle upon Tyne Hospitals	Newcastle upon Tyne	UK

Clinician	Hospital	City	Country
R Hennekam	Great Ormond Street Hospital for Children	London	UK
S Holden	Cambridge University Hospitals, Addenbrooke's hospital	Cambridge	UK
S Holder	North West Thames Regional Genetic Service	Harrow	UK
T Homfray	St George's University of London	London	UK
J Hurst	Great Ormond Street Hospital for Children	London	UK
A Ionnides	Guy's and St Thomas' Hospital	London	UK
J Jarvis	Birmingham Women's Hospital	Birmingham	UK
D S Johnson	Sheffield Children's Hospital	Sheffield	UK
E Jones	The Newcastle upon Tyne Hospitals	Newcastle upon Tyne	UK
L Jones	Central Manchester University Hospitals	Manchester	UK
L Jones	The Newcastle upon Tyne Hospitals	Newcastle upon Tyne	UK
M Jongmans	Radboud University Medical Centre	Nijmegen	The Netherlands
D Josifova	Guy's and St Thomas' Hospital	London	UK
S Joss	NHS Greater Glasgow and Clyde	Glasgow	UK
J Kenny	Great Ormond Street Hospital for Children	London	UK
B Kerr	Central Manchester University Hospitals	Manchester	UK
H Kingston	Central Manchester University Hospitals	Manchester	UK
U Kini	Oxford University Hospitals NHS Trust	Oxford	UK
E Kivuva	Peninsula Clinical Genetics Service	Devon & Exeter	UK
F Kooy	University of Antwerp	Antwerp	Belgium
A Kraus	The Leeds Teaching Hospitals	Leeds	UK
M Kurian	Great Ormond Street Hospital for Children	London	UK
K Lachlan	University Hospital Southampton	Southampton	UK
W Lam	University of Edinburgh Western General Hospital	Edinburgh	UK
M Lees	Great Ormond Street Hospital for Children	London	UK
S Lindsay	The Newcastle upon Tyne Hospitals	Newcastle upon Tyne	UK
C Longman	NHS Greater Glasgow and Clyde	Glasgow	UK
S Lynch	Our Lady's Hospital for Sick Children	Dublin	UK
A Magee	Belfast City Hospital Trust, Northern Ireland regional Genetics Centre	Belfast	UK

Clinician	Hospital	City	Country
L Van Maldergem	Institut de Pathologie et de Génétique	Loverval	Belgium
A Male	Great Ormond Street Hospital for Children	London	UK
F Mari	University of Siena	Siena	Italy
V McConnell	Belfast City Hospital Trust, Northern Ireland regional Genetics Centre	Belfast	UK
A McGeb	Belfast City Hospital Trust, Northern Ireland regional Genetics Centre	Belfast	UK
S McKee	Belfast City Hospital Trust, Northern Ireland regional Genetics Centre	Belfast	UK
C McKeown	Birmingham Women's Hospital	Birmingham	UK
C McWilliam	Royal Hospital for Sick Children	Glasgow	UK
A Medeira	Hospital de Santa Maria	Lisbon	Portugal
S Mehta	Cambridge University Hospitals, Addenbrooke's hospital	Cambridge	UK
K Metcalfe	Central Manchester University Hospitals	Manchester	UK
S Mohammed	Guy's and St Thomas' Hospital	London	UK
J Morton	Birmingham Women's Hospital	Birmingham	UK
V Murday	NHS Greater Glasgow and Clyde	Glasgow	UK
R Newbury-Ecob	University Hospitals Bristol, NHS Foundation Trust, St Michael's Hospital	Bristol	UK
S Nik-Zainal	Cambridge University Hospitals, Addenbrooke's hospital	Cambridge	UK
A Norman	Birmingham Women's Hospital	Birmingham	UK
SM Park	Cambridge University Hospitals, Addenbrooke's hospital	Cambridge	UK
MJ Parker	Sheffield Children's Hospital	Sheffield	UK
K Prescott	The Leeds Teaching Hospitals	Leeds	UK
S Price	Oxford University Hospitals NHS Trust	Oxford	UK
A Procter	University Hospital of Wales	Cardif	UK
O Quarrell	Sheffield Children's Hospital	Sheffield	UK
J Rankin	Peninsula Clinical Genetics Service	Devon & Exeter	UK
L Raymond	Cambridge University Hospitals, Addenbrooke's hospital	Cambridge	UK
G Rea	Belfast City Hospital Trust, Northern Ireland regional Genetics Centre	Belfast	UK
W Reardon	Our Lady's Hospital for Sick Children	Dublin	UK
A Renieri	University of Siena; Azienda Ospedaliera Universitaria Senese	Siena	Italy
L Robert	Guy's and St Thomas' Hospital	London	UK
E Rosser	Great Ormond Street Hospital for Children	London	UK

Clinician	Hospital	City	Country
R Sandford	Cambridge University Hospitals, Addenbrooke's hospital	Cambridge	UK
C Schwartz	Greenwood Genetic Center	Greenwood	USA
R Scott	Great Ormond Street Hospital for Children	London	UK
I Scurr	University Hospitals Bristol, NHS Foundation Trust, St Michael's Hospital	Bristol	UK
G Senger	Kompetenzzentrum für Humangenetik	Regensburg	Germany
S Sharif	Birmingham Women's Hospital	Birmingham	UK
A Shaw	Guy's and St Thomas' Hospital	London	UK
C Shaw	Guy's and St Thomas' Hospital	London	UK
D Shears	Oxford University Hospitals NHS Trust	Oxford	UK
S Smithson	University Hospitals Bristol, NHS Foundation Trust, St Michael's Hospital	Bristol	UK
M Splitt	The Newcastle upon Tyne Hospitals	Newcastle upon Tyne	UK
R Stevenson	Greenwood Genetic Center	Greenwood	USA
A Stewart	The Newcastle upon Tyne Hospitals	Newcastle upon Tyne	UK
F Stewart	Belfast City Hospital Trust, Northern Ireland regional Genetics Centre	Belfast	UK
H Stewart	Oxford University Hospitals NHS Trust	Oxford	UK
M Suri	Nottingham University Hospital NHS Trust	Nottingham	UK
E Sweeney	Liverpool Women's NHS Foundation Trust	Liverpool	UK
S Taffinder	Great Ormond Street Hospital for Children	London	UK
G Tanteles	Nottingham University Hospital NHS Trust	Nottingham	UK
MI Tejada	Molecular Genetics Laboratory	Barakaldo	Spain
K Temple	University Hospital Southampton	Southampton	UK
J Thompson	The Leeds Teaching Hospitals	Leeds	UK
J Tocher	Sheffield Children's Hospital	Sheffield	UK
S Tomkins	University Hospitals Bristol, NHS Foundation Trust, St Michael's Hospital	Bristol	UK
C Turner	Peninsula Clinical Genetics Service	Devon & Exeter	UK
P Turnpenny	Peninsula Clinical Genetics Service	Devon & Exeter	UK
A Vanderstein	North West Thames Regional Genetic Service	Harrow	UK

Clinician	Hospital	City	Country
P Vasudevan	University Hospitals of Leicester NHS Trust	Leicester	UK
L Villard	Aix Marseille Université	Marseille	France
L Visser	Radboud University Medical Centre	Nijmegen	The Netherlands
E Wakeling	North West Thames Regional Genetic Service	Harrow	UK
A Weber	Liverpool Women's NHS Foundation Trust	Liverpool	UK
D Williams	Birmingham Women's Hospital	Birmingham	UK
L Wilson	Great Ormond Street Hospital for Children	London	UK
G Woods	Cambridge University Hospitals, Addenbrooke's hospital	Cambridge	UK
M Wright	The Newcastle upon Tyne Hospitals	Newcastle upon Tyne	UK
K Writzl	Institute of Medical Genetics, University Medical Centre	Ljubljana	Slovenia
L Yates	The Newcastle upon Tyne Hospitals	Newcastle upon Tyne	UK

Supp. References

Adzhubei IA, Schmidt S, Peshkin L, Ramensky VE, Gerasimova A, Bork P, Kondrashov AS, Sunyaev SR. 2010. A method and server for predicting damaging missense mutations. Nat Methods 7:248-249.

Altshuler DM, Gibbs RA, Peltonen L, Altshuler DM, Gibbs RA, Peltonen L, Dermitzakis E, Schaffner SF, Yu F, Peltonen L, Dermitzakis E, Bonnen PE, et al. 2010. Integrating common and rare genetic variation in diverse human populations. Nature 467:52-58.

Clifford RJ, Edmonson MN, Nguyen C, Buetow KH. 2004. Large-scale analysis of non-synonymous coding region single nucleotide polymorphisms. Bioinformatics 20:1006-1014.

Cooper GM, Stone EA, Asimenos G, Green ED, Batzoglou S, Sidow A. 2005. Distribution and intensity of constraint in mammalian genomic sequence. Genome Res 15:901-913.

Gilissen C, Hehir-Kwa JY, Thung DT, Van De Vorst M, Van Bon BW, Willemsen MH, Kwint M, Janssen IM, Hoischen A, Schenck A, Leach R, Klein R, et al. 2014. Genome sequencing identifies major causes of severe intellectual disability. Nature 511:344-347.

Kumar P, Henikoff S, Ng PC. 2009. Predicting the effects of coding non-synonymous variants on protein function using the SIFT algorithm. Nat Protoc 4:1073-1081.

Stenson PD, Mort M, Ball EV, Shaw K, Phillips A, Cooper DN. 2014. The Human Gene Mutation Database: building a comprehensive mutation repository for clinical and molecular genetics, diagnostic testing and personalized genomic medicine. Hum Genet 133:1-9.

Tabor HK, Auer PL, Jamal SM, Chong JX, Yu JH, Gordon AS, Graubert TA, O'donnell CJ, Rich SS, Nickerson DA, Bamshad MJ. 2014. Pathogenic variants for mendelian and complex traits in exomes of 6,517 European and african americans: implications for the return of incidental results. Am J Hum Genet 95:183-193.

The Deciphering Developmental Disorders Study. 2014. Large-scale discovery of novel genetic causes of developmental disorders. Nature 519:223-228.

Wildeman M, Van Ophuizen E, Den Dunnen JT, Taschner PE. 2008. Improving sequence variant descriptions in mutation databases and literature using the Mutalyzer sequence variation nomenclature checker. Hum Mutat 29:6-13.
